# Multifaceted Role of PRDM Proteins in Human Cancer

**DOI:** 10.3390/ijms21072648

**Published:** 2020-04-10

**Authors:** Amelia Casamassimi, Monica Rienzo, Erika Di Zazzo, Anna Sorrentino, Donatella Fiore, Maria Chiara Proto, Bruno Moncharmont, Patrizia Gazzerro, Maurizio Bifulco, Ciro Abbondanza

**Affiliations:** 1 Department of Precision Medicine, University of Campania “Luigi Vanvitelli”, Via L. De Crecchio, 80138 Naples, Italy; erika.dizazzo@unimol.it (E.D.Z.); annasorrentino86@gmail.com (A.S.); 2 Department of Environmental, Biological, and Pharmaceutical Sciences and Technologies, University of Campania “Luigi Vanvitelli”, 81100 Caserta, Italy; monica.rienzo@unicampania.it; 3 Department of Medicine and Health Sciences “V. Tiberio”, University of Molise, 86100 Campobasso, Italy; moncharmont@unimol.it; 4 Department of Pharmacy, University of Salerno, 84084 Fisciano (SA), Italy; dfiore@unisa.it (D.F.); maproto@unisa.it (M.C.P.); pgazzerro@unisa.it (P.G.); 5 Department of Molecular Medicine and Medical Biotechnologies, University of Naples “Federico II”, 80131 Naples, Italy; maubiful@unina.it

**Keywords:** PRD-BF1 and RIZ homology domain containing gene family, human malignancies, The Cancer Genome Atlas, genetic alterations, prognosis and therapy

## Abstract

The PR/SET domain family (PRDM) comprise a family of genes whose protein products share a conserved N-terminal PR [PRDI-BF1 (positive regulatory domain I-binding factor 1) and RIZ1 (retinoblastoma protein-interacting zinc finger gene 1)] homologous domain structurally and functionally similar to the catalytic SET [Su(var)3-9, enhancer-of-zeste and trithorax] domain of histone methyltransferases (HMTs). These genes are involved in epigenetic regulation of gene expression through their intrinsic HMTase activity or via interactions with other chromatin modifying enzymes. In this way they control a broad spectrum of biological processes, including proliferation and differentiation control, cell cycle progression, and maintenance of immune cell homeostasis. In cancer, tumor-specific dysfunctions of *PRDM* genes alter their expression by genetic and/or epigenetic modifications. A common characteristic of most *PRDM* genes is to encode for two main molecular variants with or without the PR domain. They are generated by either alternative splicing or alternative use of different promoters and play opposite roles, particularly in cancer where their imbalance can be often observed. In this scenario, PRDM proteins are involved in cancer onset, invasion, and metastasis and their altered expression is related to poor prognosis and clinical outcome. These functions strongly suggest their potential use in cancer management as diagnostic or prognostic tools and as new targets of therapeutic intervention.

## 1. Introduction

The PRDM [PRDI-BF1 (positive regulatory domain I-binding factor 1) and RIZ1 (retinoblastoma protein-interacting zinc finger gene 1) homologous domain containing] gene family is a conserved subfamily of Kruppel-like zinc finger gene products, which share a conserved N-terminal PR (PRDI-BF1-RIZ1 homologous) domain, initially identified in two proteins: PRDI-BF1, currently named PRDM1 and RIZ1, now called PRDM2 [1,2,3]. Currently, 17 members are reported in humans even though two further members are considered as belonging to this family: zinc finger protein, FOG (friend of GATA-1) family member 1 (*ZFPM1*; *FOG1*) and zinc finger protein, FOG family member 2 (*ZFPM2*; *FOG2*) [2,4]. The PR domain is a subtype of the SET [Su(var)3-9, enhancer-of-zeste and trithorax] domain that defines a large group of histone methyltransferases (HMTs), with some of them functioning as lysine methyltransferases (KMTs) [1,2,3]. Enzymatic activity has been established only for a few family members [1,2,3,5,6,7]. Otherwise, PRDM proteins (PRDMs) lacking intrinsic activity are able to directly or indirectly recruit and interact with histone-modifying enzymes to positively or negatively regulate chromatin structure; these enzymes include HMTs, PRMT5 (protein methyltransferase 5), LSD1 (lysine specific demethylase 1, KDM1A), histone deacetylases (HDACs), and histone acetyltransferases (HATs) [1,2,3]. These molecular features imply their involvement in epigenetic regulation of gene expression [1,2,3].

With rare exceptions, the PR domain is generally positioned at the N-terminal region of the protein, and it is followed by repeated zinc fingers that mediate sequence-specific DNA binding and protein–protein interactions or play an important role in nuclear import [1,2,3,8,9]. This capability provides a high level of complexity and specificity. PRDM proteins connect transcription factors to target DNA promoters through the recognition of specific consensus sequences or acting as non-DNA binding cofactors [9,10]. So far, specific consensus DNA sequences and binding partners have been fully characterized only in a subset of the family members [1,2,3]. Of note, PRDMs show strong context dependency through selection of different target promoters, binding sites, and partners [1,2,3]. Overall, these features contribute to their capability of participating in many developmental processes. Significant examples are PRDM14, which is essential for pluripotency in embryonic stem cells [11] and PRDM16, which is an important player in lipid metabolism, adipocyte differentiation, hematopoiesis, and cardiac development [10,12]. In addition, PRDMs are involved in the transduction of many signals controlling cell fate and homeostasis [1]. Overall, based on PRDMs molecular structure and functions, it is conceivable that their alteration may have a pivotal role in many human diseases, including cancer. Some evidence suggests that PRDMs are involved in human malignancy through modulation of several processes such as epigenetic modifications, genetic reprogramming, inflammation, and metabolic homeostasis.

A common characteristic of most *PRDM* genes is to express two main molecular variants, one lacking the PR domain (*PR*− isoform) but otherwise identical to the other *PR+* product. These two isoforms, generated by either alternative splicing or alternative use of different promoters, play opposite roles, particularly in cancer [1,2,3]. Specifically, the full-length product usually acts as a tumor suppressor, whereas the short isoform functions as an oncogene. The imbalance in favor of *PR*− is observed in many human malignancies and it can be due to inactivating mutations or silencing of the complete form and/or to increased expression of the *PR*− form [2,3].

Here, we summarize the current knowledge on *PRDM* genes and their products by focusing mainly on their relationships with oncogenesis. Moreover, we attempt to provide insights for the future use of PRDMs as diagnostic biomarkers or therapeutic targets, by directly affecting their intrinsic catalytic activities, or by indirectly affecting their regulated pathways.

## 2. Role of PRDM Genes in Cancer

An overview of cancer-specific alterations affecting PRDM family members, taking into account putative causes, produced effects, and underlying molecular mechanisms, is detailed below and summarized in Table 1.

### 2.1. PRDM1

PRDM1/BLIMP1 (B lymphocyte-induced maturation protein-1) was firstly identified as a repressor of human β-interferon gene expression [13]. Then, it appeared as a pleiotropic regulatory factor participating at the B lymphocyte terminal differentiation [14]. PRDM1/BLIMP1 is also expressed in T and NK (natural killer) cells where it regulates their homeostasis [15,16,17]. The human gene is localized on chromosome 6q21-q22.1, a locus frequently deleted in lymphoid tumors. It encodes for a transcription repressor and it is a well-established tumor suppressor gene in human DLBCL (diffuse large B cell lymphoma) and in other hematological malignancies [17,18]. Initially, in 2006, structural alterations inactivating PRDM1/BLIMP1 were identified in DLBCLs in two independent studies [19,20]. Since then, various evidence has indicated that *PRDM1/BLIMP1* acts as a tumor suppressor gene in different types of lymphomas derived from B, T, and NK cells, and has a role in the pathogenesis of these diseases [18,21,22,23,24,25,26,27]. Particularly, disruption of *PRDM1/BLIMP1* function is frequently observed in the activated B-cell-like (ABC) subtype of DLBCL by distinct mechanisms including inactivating mutations, chromosomal deletion, and epigenetic silencing [20,24,25]. Of note, a more recent study demonstrated that its genetic loss could contribute to the overall poor prognosis for ABC-DLBCL but not germinal center B-cell-like (GCB)-DLBCLs. Furthermore, the lack of *BLIMP1* expression correlated with an impaired p53 signaling pathway and Myc overexpression; gene expression profiling data also indicated that inactivated *BLIMP1* could facilitate DLBCL progression through Myc and BCR (B cell receptor) signaling, which are essential for ABC-DLBCL survival [26]. Its inactivation was also found to be mutually exclusive with B cell lymphoma (BCL)6 alterations thus suggesting a further mechanism of transcriptional repression by constitutively active BCL6 [27]. Its involvement in these malignancies is also corroborated by both functional studies and mouse models; indeed, conditional deletion of Prdm1 in mouse B cells induced the activation of B cells with enhanced proliferative capacity. These cells failed to undergo terminal differentiation, because of the altered expression regulation of genes relevant for cell cycle progression [27]. In addition, PRDM1 ectopic expression in a DLBCL-derived cell line triggered cell cycle arrest [27]. Interestingly, this result was also achieved in other cellular settings [28]. Nevertheless, since Prdm1-null mice exhibited a long latency of lymphomagenesis, the requirement of additional oncogenic hits for DLBCL development was suggested. Consistently, an in vivo study showed that mouse Prdm1 deletion cooperated with constitutive activation of the NF-κB pathway to support a neoplastic phenotype [29].

Recent high-throughput molecular and genomic profiling analyses have significantly contributed to the understanding of the molecular basis of T and NK cell lymphomas. For instance, array comparative genomic hybridization and gene expression profiling in extranodal NK/T-cell lymphoma (EN-NK/T) revealed that the most frequently deleted chromosomal region 6q21-6q25, induced a downregulation of several tumor-suppressor genes including *PRDM1* [17,30]. Once again, its inactivation might be also due to *PRDM1* gene mutation, aberrant miRNA upregulation, and/or other epigenetic changes such as hypermethylation [31,32]. Notably, PRDM1 expression exerted an effect on the patient outcome [30,32,33]. Thus, PRDM1 expression could be endowed with an important clinical prognostic value, and its inactivation could be an important pathogenetic mechanism for EN-NK/T-NT (nasal type). Accordingly, a study employing a semi-quantitative RT-PCR assay showed that the average PRDM1 expression levels in neoplastic samples were significantly lower than those in normal NK cells used as control [32]. Likewise, PRDM1 expression was related with the stage of the disease and had a positive effect on prognosis [32]. A more recent analysis revealed a low PRDM1 expression in the majority of EN-NK/T-NT cases, and established the effect of PRDM1 expression on the prognosis of this malignancy [30].

PRDM1 does not have an intrinsic methyltransferase activity and exerts its functions through interaction with HDACs and G9a or by directly binding and repressing Myc transcription factor [34,35]. *PRDM1* mutations occurred in patients with plasmablastic lymphoma; interestingly, in this rare neoplasm, *PRDM1* genetic alterations did not impair terminal B-cell differentiation, but contributed to the oncogenicity of MYC, which is usually dysregulated by translocation or amplification. Aberrant co-expression of MYC and the full-length isoform of PRDM1, PRDM1α, due to genetic changes was responsible for the phenotype of plasmablastic lymphoma cases [36]. This is in accordance with the study on ABC-DLBCL patients where the lack of BLIMP1 expression correlated with Myc overexpression [26].

**Figure 1:**
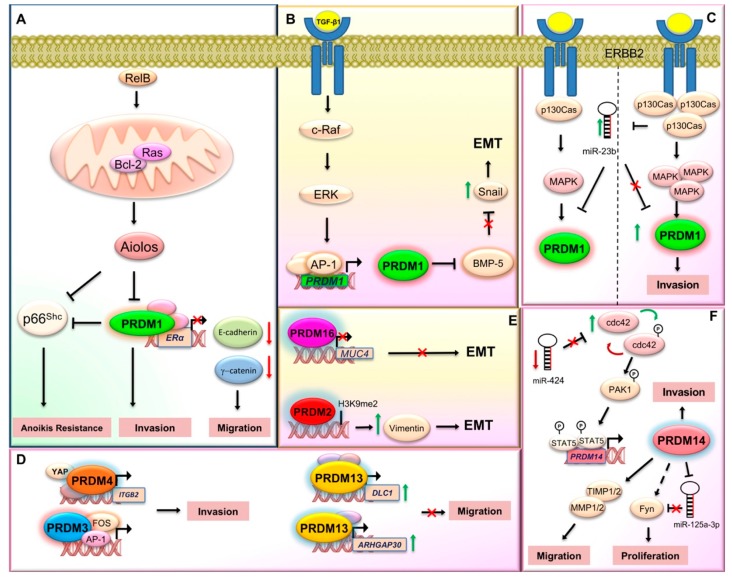
PRDM proteins contribution in the mechanisms related to invasiveness and metastasis. This scheme illustrates the proposed molecular mechanisms involving some PRDMs during invasion and metastasis. (**A**) Higher PRDM1 expression is detected in estrogen receptor alpha (ERα)-negative breast cancer cells and primary breast tumors. Mechanistically, Bcl-2, induced by RelB, interacts with and activates Ras in the mitochondrial membrane. In turn, Ras induces the expression of PRDM1/BLIMP1, which downregulates ERα gene expression by direct binding to its promoter, thus promoting a reduction in the levels of E-cadherin and γ-catenin and a corresponding increase in the migratory phenotype of breast cancer cells. The lymphocyte lineage-restricted transcription factor Aiolos negatively regulates PRDM1 and p66^Shc^ transcription; in addition, loss of PRDM1 expression reduces the expression of p66^Shc^. Thus, the absence of PRDM1 protein promotes cancer cell invasion and at the same time confers anoikis resistance to the cancer cell. (**B**) TGF-β1 promotes PRDM1/BLIMP1 gene transcription via c-Raf and AP-1 pathway. Blimp1, in turn, by reducing the expression of BMP-5, induces the expression of SNAI1, the epithelial–mesenchymal transition (EMT) master regulator. (**C**) The miR-23b downmodulation and the ErbB2/p130Cas/MAPK axis activation increases the expression of the transcriptional repressor PRDM1/Blimp1, thus mediating cell invasion. (**D**) PRDM3 synergizes with FOS in expression regulation of gene products controlling cell invasion. PRDM4 mediates cell invasion by interacting with YAP at *ITGB2* gene promoter. PRDM13 upregulates DLC1 and ARHGAP30 proteins thus inhibiting cell invasion. (**E**) PRDM2 controls the expression of several genes involved in EMT, with vimentin being the most significantly regulated gene. PRDM16 inhibits EMT by repressing the transcription of *MUC4*. (**F**) The miR-424→cdc42→prdm14 axis controls cell invasion. In particular, miR-424 knockdown induces expression of Cdc42 that in turn positively regulates PRDM14 through the activation of Pak1 and Stat5. PRDM14 promotes cell migration by regulating the expression level of matrix metalloproteinase (MMP)/tissue inhibitor of metalloproteinases (TIMP). Knockdown of PRDM14 reduced cancer stem cell phenotypes via miR-125a-3p and Fyn expression regulation in pancreatic cancer (see text for additional details).

Altered expression of PRDM1 has also been investigated in several non-hematopoietic cancer cells. For instance, higher PRDM1 expression was detected in estrogen receptor alpha (ERα)-negative breast cancer cells and primary breast tumors [37]. Mechanistically, B cell lymphoma (Bcl)-2, induced by RelB, interacts with and activates Ras in the mitochondrial membrane. The activation of Bcl-2/Ras pathway leads to the expression of *PRDM1/BLIMP1* gene. In turn, PRDM1/BLIMP1 downregulates ERα gene expression by direct binding to its promoter, thus promoting a reduction in the levels of E-cadherin and γ-catenin and a corresponding increase in migratory phenotype of breast cancer cells (Figure 1A) [37]. Interestingly, a later study demonstrated that PRDM1/BLIMP1 played an essential role in transforming growth factor (TGF)-β1-induced epithelial–mesenchymal transition (EMT) signature and cell migration of breast cancer cells via BMP-5 repression (Figure 1B) [38]. Additionally, both in vitro and in vivo models have recently described the role of PRDM1/Blimp1 in p130Cas/ErbB2 breast cancer invasion; particularly, Blimp1 was highly expressed during p130Cas/ErbB2 dependent invasion in breast cancer cells and the downregulation of its expression was sufficient to severely impair tumor invasiveness in vitro and lung metastasis formation in vivo [39]. In addition, PRDM1 expression positively correlates with a higher probability of developing multiple metastasis in patients with high p130Cas, supporting the major role played by p130Cas in Blimp1-mediated invasion [39]. Additionally, this study showed that Blimp1 expression was negatively regulated by mir-23b, suggesting a role as a tumor suppressor for this miRNA in p130Cas/ErbB2 cells. Altogether, the miR-23b impairment and the ErbB2/p130Cas/MAPK axis activation could increase PRDM1/Blimp1 expression level, thus mediating cell invasion (Figure 1C) [39].

**Figure 2:**
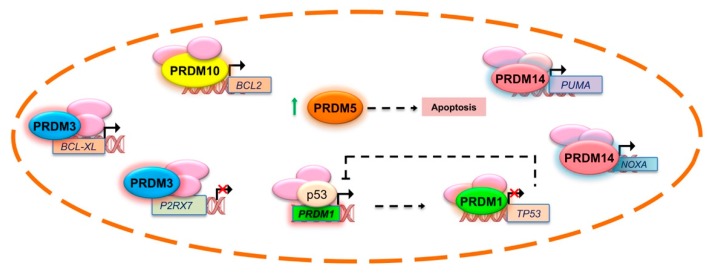
PRDM proteins action in the regulation of apoptosis genes expression. Although the precise and direct involvement of PRDMs in apoptosis is not completely unravelled, it is established that they are able to control the expression of several genes participating in this biological process, like *BCL-XL*, *BCL2*, and *TP53* among the others. This scheme illustrates the regulation of apoptotic genes by PRDMs where a direct link was demonstrated (see text for additional details).

In colorectal tumor cells, PRDM1 knockdown by small-interfering RNA (siRNA) results in both apoptosis and growth arrest through regulation of p53 transcription. Interestingly, both p53 mRNA and protein levels are considerably increased after PRDM1/BLIMP1 depletion, which is accompanied by the induction of p53 target genes. Thus, PRDM1/BLIMP1 binds to the *TP53* promoter and represses its transcription. p53, in turn, binds to and positively regulates *PRDM1/BLIMP1* (Figure 2) [40]. As for other members of the PRDM family, the existence of an alternative protein product of the *PRDM1* gene was previously described in myeloma cell lines; this protein, named PRDM1β, is generated by alternative transcription initiation using an internal promoter and it has a disrupted PR domain and lacks the amino-terminal 101 aa of the originally described protein [41]. Noteworthy, a recent study showed that PRDM1β was a p53-response gene in human colon organoids and low PRDM1 expression could predict poor survival in colon cancer patients [42]. However, today the functional role of these protein isoforms still needs to be elucidated.

**Figure 3:**
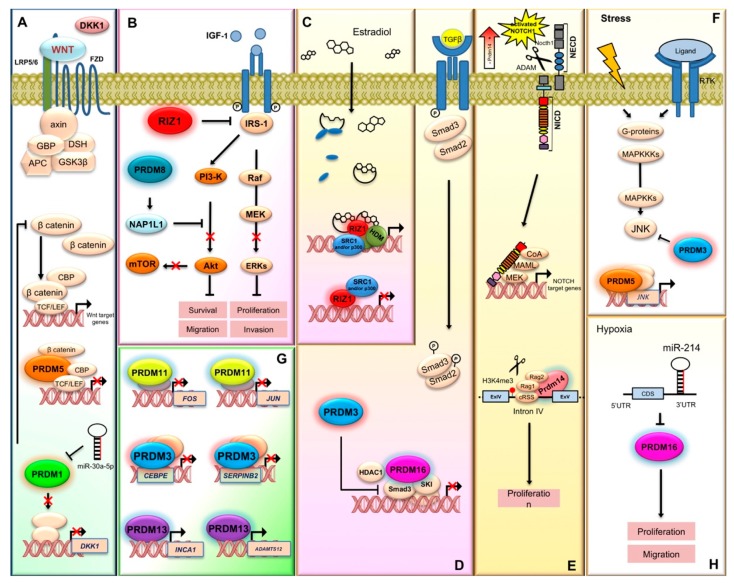
PRDM proteins participation in signal transduction pathways, proliferation, and gene expression regulation. PRDM proteins play a pivotal role in the transduction of signals that control cell proliferation and differentiation. (**A**) PRDM1 and PRDM5 antagonize the Wnt/β-catenin pathway. PRDM1 reduces the expression of *DKK1* while PRDM5 forms a chromatin complex with CBP, TCF, and β-catenin that prevents Wnt target gene expression. (**B**) PRDM2/RIZ1 counteracts the insulin-like growth factor-1 (IGF-1) receptor and the downstream signaling component ERK1/2 and AKT. PRDM8 suppresses the PI3K/AKT/mTOR signaling cascade through the regulation of nucleosome assembly protein 1-like 1 (NAP1L1). (**C**) The *PRDM2* gene product, PRDM2a/RIZ1, is a downstream effector of estrogen action and is related to estrogen-regulated cancer cell proliferation. ERα modulates the PRDM2/RIZ isoforms intracellular concentration ratio, by an indirect and selective decrease of RIZ1 expression and a transcriptional activation of RIZ2. (**D**) TGF-β signaling plays important roles in cytostasis and normal epithelium differentiation, and alterations in TGF-β signaling have been identified in many malignancies. MEL1/PRDM16 interacts with SKI and inhibits TGF-β signaling by stabilizing the inactive Smad3-SKI complex on the promoter of TGF-β target genes. PRDM3 negatively regulates TGF-β signaling through binding and inactivating SMAD3 proteins. (E) PRDM14 binds an intron of *NOTCH1* gene and modifies the chromatin structure (H3K4me3) allowing access of the RAG recombinase complex. RAG deletes part of the *NOTCH1* promoter and consequently a truncated, ligand-independent Notch1 protein is produced. (**F**) PRDM3 through its first zinc finger domain, associates and inhibits JNK activity, thus protecting cells from stress-induced cell death that is dependent on JNK activation. Otherwise, PRDM5 upregulates JNK expression. (**G**) PRDM11 represses the oncogenes Fos and Jun that are frequently induced by aberrant growth factor signaling or oncogenic activation of MAP kinase signaling, such as constitutively active RAS. PRDM3 downregulates *SERPIN-B2* gene that might play an important role in enhancing cell proliferation by preventing protection of Rb proteolysis and/or in the suppression of cell differentiation. PRDM13 inhibits cell proliferation by upregulating INCA1, a CDK inhibitor and ADAMTS12, a novel antitumor protease that modulates the extracellular signal-regulated kinase signaling pathway. (**H**) Hypoxia-induced miR-214 inhibits *PRDM16* expression, thus promoting both cell proliferation and migration and enhancing the Warburg effect.

PRDM1 is also associated with glioma malignancy [43]. Indeed, PRDM1 expression levels decreased significantly with ascending glioma grade and correlated positively with Dickkopf-1 LDL low-density lipoprotein (DKK1) levels. In addition, PRDM1 reduced the expression of *DKK1* thus exerting its antitumor effect via antagonizing the activity of Wnt/β-catenin pathway (Figure 3A) [43].

Reduced expression of PRDM1 has also been associated with poor prognosis in lung cancer where it can promote cellular invasion and anoikis resistance in vitro and metastasis in vivo. Specifically, the ectopic expression of the transcription factor Aiolos induced anoikis resistance to cancer cells by downregulating PRDM1. The transcription of these two genes was negatively correlated in 206 lung epithelial cell lines and reduced the expression of p66^Shc^. Thus, PRDM1 deprivation induced cancer metastasis through cell invasion promotion and anoikis resistance through p66Shc transcription decrease (Figure 1A) [44].

Interestingly, our recent pan-cancer analysis of The Cancer Genome Atlas (TCGA) datasets also confirmed that *PRDM1* gene is often genetically altered in DLBCL, with 8.2% mutation frequency [45]. Additionally, mutations were revealed in some solid tumors, such as skin cutaneous melanoma and uterine carcinosarcoma, which displayed more than 5% of patients carrying *PRDM1* mutations [45].

Overall, these literature data established a key role for this gene and its protein products in both hematological and solid malignancies; more importantly, they also provided the mechanisms to target them in cancer therapy. Indeed, recent findings suggest that PRDM1/BLIMP1 expression could be restored through the use of pan-HDAC inhibitors like vorinostat [46].

### 2.2. PRDM2

*PRDM2* is localized in a chromosome region (1p36), which is commonly affected by genetic alterations in a broad range of human malignancies thus suggesting a tumor-suppressor role for this gene ([3] and references therein). Two main protein products, known as RIZ1 (PR+) and RIZ2 (PR−), with and without the PR domain, are generated by an internal promoter [47]. As proposed for other PRDMs, their imbalance may constitute an important cause of malignancy with the PR+ product commonly lost or downregulated and the PR− isoform always present at higher levels in cancer cells [48,49,50]. Indeed, both genetic inactivation or epigenetic silencing of RIZ1 and/or an increase of RIZ2 expression levels were frequently revealed in many human cancer tissues and cell lines [45,51]. This observation suggested that RIZ1 could negatively regulate cell growth and tumorigenesis whereas RIZ2 could be necessary for oncogenesis by promoting cell proliferation through its mitogenic activity [52,53]. RIZ2 oncogenic properties were linked to the first cluster of Zn-fingers since stably transfected MCF-7 cells, in both estrogen deprivation or stimulation conditions, showed an increased proliferation compared to control cells and reduced responsiveness to the growth inhibitory effect of anti-estrogens, conceivably due to the altered expression of proteins involved in cell proliferation and differentiation [53,54]. However, the functional difference in chromatin structure regulation between RIZ proteins, through the PR domain, may also account for their opposite roles in tumorigenesis [8]. *Riz1* knockout mice, carrying normal *Riz2*, were tumor prone in both wild-type and mutant *p53* genetic backgrounds. Indeed, an accelerated tumorigenesis was associated with Riz1 deficiency (Riz1^-/-^) on the p53^+/-^ background [55]. Interestingly, this Riz1-p53 cooperation was also found in many human tumors [51,55].

Several studies have established a role for *PRDM2* in tumors that acquire chromosomal instability (CIN) [56,57]. Indeed, frameshift mutations of microsatellite repeats localized in the C-terminal coding region were frequently detected in colorectal, gastric, endometrial, and pancreatic microsatellite instability (MIN) positive cancers [56,57,58,59]. Mostly, these mutations were 1- or 2-bp deletions in two coding poly-adenosine tracts of *PRDM2* gene [56,57,58,59]. In a recent TCGA analysis, a somatic frameshift mutation in the (A)9 tract was found as a microsatellite indel driver hotspot in 48% of stomach tumors [60]. This finding suggested for the first time a role for *PRDM2* as a cancer driver gene [60]. Despite their high occurrence, the functional significance in tumorigenesis of these C-terminal *PRDM2* truncated forms induced by frameshift mutations is still unknown and deserves investigation. Interestingly, the restoration of the wild-type *PRDM2* gene sequence of one mutant c.4467delA allele by genome editing in homozygous mutant human colorectal cancer cells, repaired its H3K9me2 activity, impaired tumor cell growth, reduced anchorage-independent growth, cellular migration, and colony forming ability in vitro, as well as decreased the tumor growth in a mouse xenograft model [61]. Furthermore, H3K9me2 activity restoration determined the downregulation of several genes involved in cancer pathways, mostly of EMT, thus contributing to a more aggressive cancer phenotype (Figure 1E) [61]. In addition, frameshift mutations in the (A)9 tract were also found in samples of malignant melanoma and nevi [62] and in leukemia cell lines [63]. Interestingly, in most cases of MIN pathway cancers these frameshift mutations were biallelic or homozygous/hemizygous, indicating that *PRDM2* follows the two-hit model of tumor suppressor genes, with one hit achieved either by mutations/deletions affecting the PR domain or by frameshift mutations in the 3′ end affecting the interactions between the N-terminal PR domain of RIZ1 and its C-terminal region, including the PR-binding motif [59]. Recently, our mutational analysis of TCGA datasets found that the *PRDM2* gene is often altered in stomach, colon, and endometrial carcinomas, with a mutation frequency higher than 5% [45].

Interestingly, some *PRDM2* polymorphisms have also been associated with carcinogenesis [3,63,64,65,66]. RIZ1 loss or its nuclear-cytoplasmic localization switch also occurred in prostate and endometrial cancer cells and tissues, with a RIZ1 staining intensity decrease from highly to poorly differentiated tumors [67,68].

A CpG island in the *PRDM2/RIZ1* promoter is frequently methylated in many cancer types, such as breast carcinomas and liver tumors, as well as in colon and lung cancer cell lines [69,70]. Additionally, epigenetic silencing of *RIZ1* expression was also detected in pituitary adenomas and nasopharyngeal carcinoma specimens [71,72]. In most of these cases, mutations in *PRDM2* gene were not detected [64,65] suggesting that DNA methylation would be the preferred mechanism of RIZ1 inactivation in these malignancies [69].

Mechanistically, the role of PRDM2 products in cancer can be explained partly through its known functions. For instance, many cancers show an increased activation of insulin-like growth factor-1 (IGF-1) signaling pathways and PRDM2a/RIZ1 is able to counteract the IGF-1 receptor and the downstream signaling cascade components ERK1/2 and AKT (Figure 3B) [73]. Furthermore, it is well documented that PRDM2/RIZ1 isoform is a downstream effector of estrogen action in target tissues (Figure 3C) [1,3,49,74,75,76,77]. Indeed, estradiol induced a proliferation increase in both estrogen-responsive cells and estrogen target tissues and this increase was correlated to a modulation of the expression of RIZ isoforms, mainly a selective decrease of RIZ1 expression, with a consequent shift in the balance of their intracellular concentrations [49,74,75,76,77]. The estrogen-induced effect on RIZ1 expression was indirect, whereas the transcriptional activation of RIZ2 was a direct estrogen receptor-mediated effect induced by estradiol [76]. Noteworthy, a later study demonstrated that estradiol induced the preferential synthesis of transcripts with exon 9a, whereas it reduced those containing exons 9b and 10 [78]. The significance and the possible different functions of these diverse RNA tails are still unknown. A possible explanation could be the presence of recognition consensus sequences for several miRNAs in the exon 9, as revealed by bioinformatics analysis, since various miRNAs are known to regulate estradiol response in breast cancer cells [79]. Additionally, estradiol could increase RIZ2 expression levels through the induction of *MYC* gene expression; indeed, *RIZ2* promoter contains a conserved canonical c-Myc binding site [76]. Interestingly, PRDM2/RIZ1 was involved in the mediation of androgen and estrogen effects also in non-classic estradiol target tissues like prostate [67]. In addition, a selective expression of PRDM2/RIZ1 was correlated with induced myeloid cell differentiation thus suggesting the role of *PRDM2* gene products in the proliferation/differentiation switch [80].

The PR domain of PRDM2/RIZ1 itself showed growth inhibitory and anticancer activities; indeed, it increased cell death when transfected in human hepatoma HuH7 cells [81]. Likewise, it inhibited cell proliferation and induced apoptosis in cultured primary meningioma cells and limited xenograft high-grade meningiomas tumor growth in nude mice through its methyltransferase activity [82]. Through this mechanism, the PR-domain modulated the expression of many genes involved in important cellular processes, including the downregulation of the *MYC* oncogene and the upregulation of the tumor suppressor thioredoxin binding protein (TXNIP), which increased reactive oxygen species production and oxidative stress, resulting in cellular apoptosis [82]. More recently, a study demonstrated that PRDM2/RIZ1 could lead to G2/M arrest of meningioma cells by acting on the expression of the checkpoint protein UbcH10, an important member of the ubiquitin-conjugating enzymes family, through c-Myc degradation [83].

**Figure 4:**
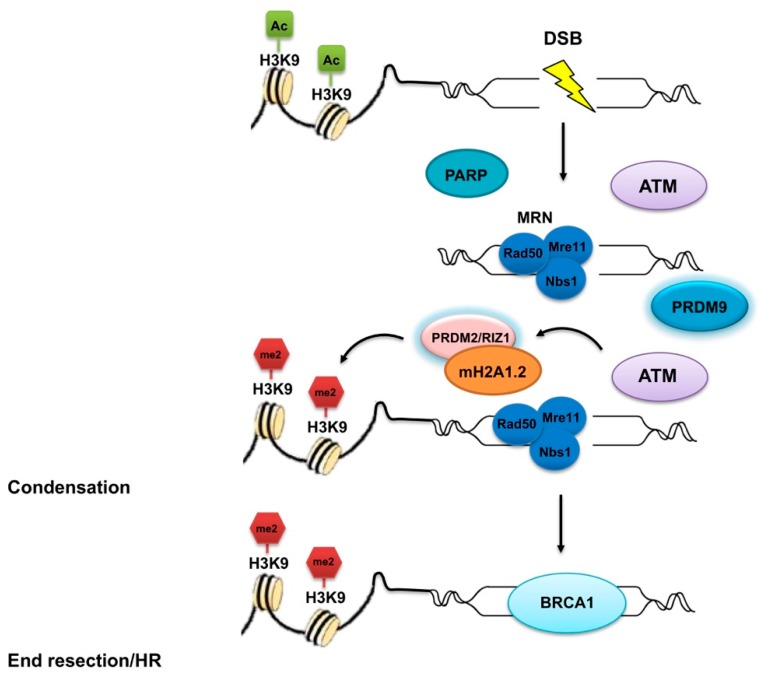
Involvement of PRDM proteins in double-strand break (DSB) DNA repair. Many insults are responsible for DNA double-strand breaks (DSBs) that impair DNA replication and proper chromosome segregation. DSB repair system disfunction is frequently observed in cancer, thus rendering cells prone to transformation. PRDM2/RIZ1 and PRDM9 are implied in the DSB repair complex, which is essential for ensuring accurate DNA repair and maintenance of genomic integrity. First, PARP is recruited at the DSB where it catalyzes the formation of poly (ADP-ribose) chains, facilitating the docking of the MRN complex to the DSB. The MRN complex, with its nuclease activity and DNA binding capability, is involved in the initial processing of DSBs. Subsequently, ataxia telangiectasia mutated (ATM) kinase induces the recruitment of the mH2A1.2/RIZ1 complex at DSB sites. PRDM2/RIZ1 induces the H3K9me2 and in that way enables a dynamic switch in chromatin conformation. Finally, the mH2A1.2/RIZ1 module recruits BRCA1. PRDM9 also affects the DSB initiation and repair, thus allowing genetic exchange between chromosomes.

For effective PRDM2/RIZ1 tumor suppressor activity, the PR-binding motif is required [84]. Specifically, tumor suppressor function requires the establishment of the H4K20me1-H3K9me1 trans-tail ‘histone code’ at specific loci through the direct interaction of RIZ1 PR-binding motif to PR-Set7 monomethyltransferase, an essential component of the mammalian cell cycle, which is needed for proper DNA replication and mitosis, thus hypothesizing an additional mechanism of action [84]. RIZ1 and PR-Set7 might cooperate in DNA replication to prevent aberrant genome duplication [84] and in mitotic chromosome segregation to prevent genomic instability [84]. Since the loss of PR-Set7 produced persistent DNA double-strand breaks (DSBs), it was conceivable that H4K20me1, and possibly Riz1-mediated H3K9me1, had a role in DNA repair [84]. Accordingly, a more recent study suggested PRDM2/RIZ1 as a component of the DSB repair complex, which is essential for ensuring accurate repair outcome and genomic integrity maintenance (Figure 4) [85]. Essentially, it would cooperate with the macrohistone variant mH2A1.2 to direct the choice between the antagonistic DSB repair mediators, BRCA1 and 53BP1. As shown in Figure 4, PARP is recruited at DSBs where it catalyzes the formation of poly (ADP-ribose) chains, facilitating the docking of an MRN complex to the DSB. The MRN complex, with its nuclease activity and DNA binding capability, is involved in the initial processing of DSBs. Subsequently, ataxia telangiectasia mutated (ATM) kinase induces the recruitment of the mH2A1.2/RIZ1 complex at DSB sites. The mH2A1/RIZ1 module enables a dynamic switch in chromatin conformation through H3K9me2 mediated by the PRDM2 methyltransferase. Then, a homologous recombination and repair through BRCA1 follows [85].

Altogether, several results indicate that RIZ1 has tumor suppressor activities, whereas RIZ2 could function as an oncogene with putative intrinsic growth-promoting properties; however, many issues deserve to be elucidated, including the underlying molecular mechanisms and the involved cellular pathways.

### 2.3. MECOM/PRDM3

*PRDM3/MECOM* (*MDS1* and *EVI1* Complex) locus was firstly identified as a site of proviral insertion in murine myeloid leukemias [86,87]. This locus is localized at chromosome band 3q26.2 and is formed by the fusion of two coding genes with two distinct transcription starting sites and isoform subgroups produced by alternative splicing events: myelodysplasia syndrome 1 (*MDS1*) and ecotropic virus integration site 1 (*EVI1*) [88,89]. Hence, the PR domain-containing product is formed by combining the two genes; the *PR*− isoform, named EVI1 or sPRDM3 (short PRDM3), can be transcribed separately [88].

It is well established that chromosomal rearrangements or proviral insertion at the *PRDM3* locus gene, *MECOM*, are found in up to 10% of acute myeloid leukemia (AML) cases with poor survival outcomes [86,90,91,92,93,94,95]. The expression levels of particular MECOM isoforms propose that the N-terminal region of PRDM3 confers a tumor suppressor function, while the shorter EVI1 isoform is overexpressed in several malignancies and could have oncogenic properties in both myeloid and solid tumors [96,97,98]. Intriguingly, in epithelial ovarian cancer, the increases in *EVI1* DNA copy number and MDS1/EVI1 transcripts were associated with improved patient outcomes, whereas EVI1 transcript levels were associated with a poor patient survival. Thus, favorable prognosis associated with increased DNA copy number derived from the high expression level of the fusion transcript MDS1/EVI1 [99]. Later, in ovarian tumors a high frequency of aberrant EVI1 splicing, generating novel isoforms, could contribute to the pathophysiology of these cancers [100].

EVI1 is usually upregulated through the generation of oncogenic fusion proteins as a consequence of rearrangements [101]; alternatively, it can also be upregulated by leukemogenic factors at the transcriptional level [102]. Although increased levels of this protein in several leukemia subtypes are well documented, data from ectopic expression of EVI1 are still weak [94]. Particularly, results from mouse models were quite variable probably due to technical differences and/or to the context-dependent oncogenic function of EVI1, which could be specific to certain hematopoietic cell types [103,104,105,106]. A further reason could be that EVI1 by itself would not be sufficient to induce a neoplastic disease [94]. Recently, an in vivo study demonstrated that EVI1 overexpression altered hematopoiesis, with suppression of erythropoiesis and lymphopoiesis, and marked expansion of myelopoiesis that eventually could result in leukemic transformation [94]. The underlying molecular mechanism involved the upregulation of Spi1, encoding the transcription factor PU.1, a master regulator of early myelopoiesis, that would drive the hematopoietic stem/progenitor cells towards the myeloid lineage [94]. In a previous study, comprehensive genome-wide EVI1 binding and whole transcriptome gene deregulation were observed in leukemic cells using a combination of ChIP-Seq and RNA-seq expression profiling [107]. This study demonstrated that EVI1 directly bound to and downregulated the master myeloid differentiation *CEBPE* gene and several of its downstream gene targets critical for terminal myeloid differentiation. Additionally, EVI1 bound to and downregulated the serine protease inhibitor (SERPIN)-B2 which might play an important role in enhancing cell proliferation by preventing protection of Rb proteolysis and/or in the suppression of cell differentiation (Figure 3G), as well as numerous genes involved in Jak-Stat signaling and in apoptosis mediated by ATP-dependent purinoreceptors [107].

Functionally, several other mechanisms have been ascribed to this oncogene. EVI1 has been implicated in the maintenance and expansion of normal hematopoietic stem cells [108,109]; particularly in the hematopoietic system, EVI1 expression is restricted to long-term and short-term hematopoietic stem cells and, under normal conditions, is transcriptionally silenced during hematopoiesis [109]. EVI1 exerts its biological effects mainly through the regulation of gene transcription by acting as a sequence specific transcription factor, by modulating the activity of other transcription factors and regulating promoter CpG island methylation [7,110]. For instance, the proximal set of EVI1 zinc fingers is able to bind the N-terminal domain of the zinc finger transcription factor hypermethylated in cancer 1 (HIC1); in turn, this interaction deregulates the DNA binding and transcriptional activity of EVI1 on the *BCL-XL* promoter, thus compromising the anti-apoptotic activity of EVI1 (Figure 2) [111].

As mentioned above, EVI1 overexpression has been observed in many solid tumors [112,113,114] and its deregulation is often associated with poor prognosis [115,116]. For instance, a study detected EVI1 overexpression in prostate cancer (PC); as observed in 10–50% of myeloid malignancies, it did not result from genetic aberrations but from a still unknown alternative mechanism [89,115]. EVI1 knockdown impaired PC cell proliferation through a cell cycle progression blockade. Mechanistically, these changes might be at least in part mediated by reactivation of SMAD3, a known transcriptional target of EVI1 [115,117]. EVI1 knockdown in PC cells also reduced migratory potential and anchorage-independent growth while enhancing apoptosis sensitivity [115]. Additionally, in a comprehensive expression and functional analysis, high EVI1 protein expression was observed in breast carcinoma where it showed a prognostic significance in ER−, and especially triple-negative, tumors [116]. EVI1 silencing reduced proliferation, apoptosis resistance, and tumorigenicity and these effects were rescued by estrogen supplementation in ER+ breast carcinoma cells. Moreover, microarray analysis identified G-protein-coupled receptor signaling as a possible effector mechanism, with *KISS1* as a novel transcriptional target of EVI1, which together promote cell migration [116].

Further evidence revealed that EVI1 might act by regulating signaling pathways that ultimately lead to increasing tumor cell proliferation and apoptosis suppression [118,119]. For instance, EVI1 protects a variety of cells from stress-induced cell death that is dependent on JNK activation. Indeed, EVI1 was able to associate with JNK through the first zinc finger domain and this association was required for efficient JNK inhibition (Figure 3F) [120]. By protecting cells from apoptotic signals, suppression of JNK activity might contribute to the oncogenic potential of EVI1 [120]. DNA-protein binding analysis revealed that EVI1 could directly bind its target genes through its zinc finger domain and regulate the expressions of different genes as *GATA2*, *GATA3*, and *ZFPM2* [121,122].

Publicly available microarray datasets showed a negative correlation between EVI1 and all the known EMT related transcription factors (SNAIL, SLUG, ZEB1, ZEB2, TWIST1, and TWIST2) in colon cancer patient samples [123]. Particularly, SLUG, a master regulator of EMT, was regulated by EVI1 through promoter binding [123]. In addition, EVI1 knockdown demonstrated its requirement for metastasis of colon cancer cells [123]. Similarly, EVI1 could play an oncogenic role also in nasopharyngeal carcinoma growth and metastasis [124]. In human ovarian cancer, an enriched fraction of EVI1 target genes were identified by genome-wide ChIP-Seq and microarray studies. More than 25% of EVI1-occupied genes contained linked EVI1 and activator protein (AP)1 DNA binding sites providing evidence for a synergistic cooperation between EVI1 and the AP1 family member FOS in the regulation of cell adhesion, proliferation, and colony formation [125]. PRDM3 synergized with FOS in the expression regulation of genes controlling cell invasion (Figure 1D).

Recent findings have suggested *MECOM* as a novel candidate gene for hereditary hematological malignancies; indeed, a novel germline mutation within the ninth zinc finger motif was reported in a family with developed myeloid malignancies [126].

As for *PRDM2* gene, a mononucleotide repeat (A7) in exon 8 of *MECOM* coding sequences was found to be a target for frameshift mutation (loss-of-function mutation) in colorectal cancers with MIN. In the same study, the authors also observed intratumor heterogeneity (an important cancer hallmark) of *MECOM* mutations in four of 16 analyzed cases (25%) [127].

Furthermore, our TCGA analysis revealed that this gene is mutated in about 20% of skin melanomas as well as is frequently altered in bladder, colon, lung, and endometrial carcinomas, in more than 5% of cases [45]. Additionally, a *MECOM* downregulation in most of the analyzed malignancies was observed [45].

### 2.4. PRDM4

Sequence tagged sitesmarker and radiation hybrid analyses mapped *PRDM4* to human chromosome 12q23-q24.1, a region putatively harboring tumor suppressor genes for ovarian, gastric, and pancreatic cancers [128]. A later study recognized an involvement of PRDM4, which was regulated by hsa-miR-373, in the gastric cancer recurrence with the potential to act as a new prognostic biomarker in predicting recurrence risk for gastric cancer patients [129]. More recently, PRDM4 was demonstrated to support yes-associated protein (YAP)-induced tumorigenesis probably via mediating the expression of other YAP target genes, which finally contribute to cell invasion and metastasis promotion. Particularly, PRDM4 mediated cell invasion by interacting with YAP at leukocyte-specific integrin β2 (*ITGB2*) gene promoter (Figure 1D) [130].

### 2.5. PRDM5

Since its first description, PRDM5 has been considered to have a tumor suppressor role. *PRDM5* is silenced in human breast, ovarian, and liver cancers by CpG island methylation of its promoter region. Moreover, its tumor suppressor function was corroborated by the G2/M arrest and apoptosis of tumor cells upon infection of a recombinant adenovirus expressing PRDM5 [131]. Later, epigenetic *PRDM5* silencing was also shown in gastric and colorectal cancers, where its ectopic expression determined a cell growth suppression [132]. Of note, *PRDM5* promoter methylation was detected in both primary colorectal and gastric cancers but not in noncancerous tissue specimens collected from areas adjacent to the tumors [132]. Additionally, a comprehensive DNA methylation profiling by bisulfite pyrosequencing, performed on normal and cancer samples after successful eradication of *Helicobacter pylori*, recognized PRDM5 as a risk factor for gastric cancer development [133]. In addition, an in vivo study demonstrated that *Prdm5* knockout mice suppressed the number of Apc(Min)-driven intestinal adenomas and regulated monoacylglycerol lipase expression [134]. Recently, a significant *PRDM5* promoter methylation was observed in *BRAF* mutant cancers of the serrated pathway whereas minimal levels of methylation were detected in the *BRAF* wild-type cancers of the traditional pathway; moreover, *PRDM5* methylation was evident in a small proportion of serrated type polyps indicating that this may be an early event in tumorigenesis [135].

**Figure 5:**
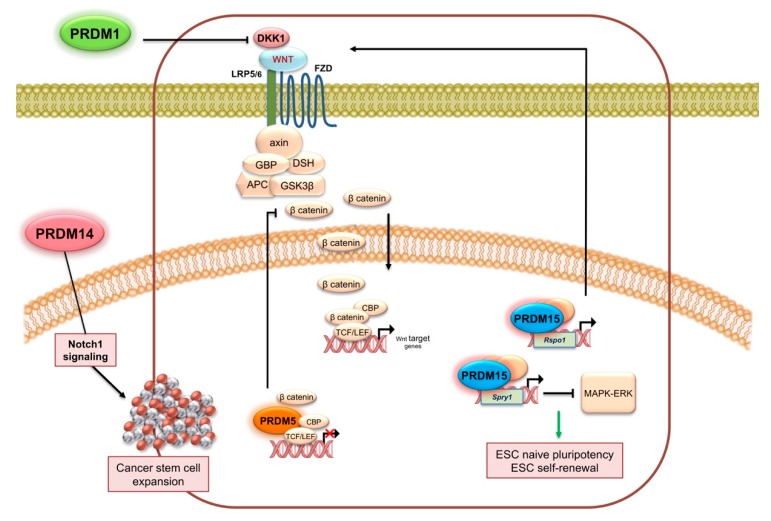
PRDM activity in cancer stemness. The figure summarizes the mechanisms regulated by some PRDMs and possibly involved in cancer stemness (see text for detailed description).

Afterwards, other literature data confirmed the proposed role of PRDM5 as a tumor suppressor since it is frequently downregulated in several malignancies. For example, aberrant DNA methylation reduced PRDM5 expression in about 40.5% of cervical cancers, whereas normal tissues were unmethylated [136]. Similarly, *PRDM5* was frequently silenced by promoter methylation in multiple cancer cell lines and tumor specimens, including nasopharyngeal, esophageal, gastric, cervical, and hepatocellular carcinoma [137]. Regarding the underlying mechanism, PRDM5 might function at least in part through negative regulation of aberrant Wnt/β-catenin signaling and oncogene expression (Figure 3A and Figure 5) [137].

Likewise, *PRDM5* promoter was methylated and its gene expression was reduced at both mRNA and protein levels in lung squamous cell carcinoma tissues. Promoter methylation significantly correlated with tumor cell differentiation and lymph node metastasis, but not with tumor grade or other parameters like age, gender, and smoking [138]. Consistently, 5-aza-2’-deoxycitydine inhibited the proliferation of the SK-MES-1 lung cancer cell line and xenograft growth in nude mice, along with reduced *PRDM5* promoter methylation and its consequent increased expression [138,139,140]. Overall, these data suggest that PRDM5 is a tumor suppressor in several human cancer types where it could represent a molecular marker for diagnosis and prognosis and a promising target for their therapy. Accordingly, this concept is also supported by a study in which repression of PRDM5 function, due to deletions in its locus along with miR-182 sequence amplification, was shown to play a co-operative role in ovarian cancers [141]. Finally, epigenome and transcriptome profiling of mouse primary liver cancer identified Tbx3 and Prdm5 as major microenvironment-dependent and epigenetically regulated lineage-commitment factors, a function that is conserved in humans [142].

However, PRDM5 could also play an opposite role in melanoma. Indeed, the results of a recent study showed that Prdm5 potentiated the progression of murine melanoma through upregulating JNK expression, suggesting that PRDM5 may function as an oncogene in this malignancy (Figure 3F) [143].

### 2.6. PRDM6

The *PRDM6* gene encodes for a transcriptional repressor involved in the regulation of endothelial cell proliferation, survival and differentiation; additionally, decreased copy numbers of this gene were found in schistosoma-associated bladder tumors [144]. Accordingly, our TCGA analysis revealed highly downregulated PRDM6 in bladder carcinomas [45]. Noteworthy, a meta-analysis of genome-wide association studies correlated a single nucleotide polymorphism in *PRDM6* gene with both mammographic density and breast cancer susceptibility [145]. “Enhancer hijacking” has been described as a mechanism in which genomic instability can lead to the utilization of an existing epigenetic structure to drive oncogene expression, for instance by repositioning a gene next to super-enhancers. Interestingly, many “enhancer hijacking” events activating PRDM6 were observed in a medulloblastoma molecular subtype [146].

### 2.7. PRDM7

In our pan-cancer reanalysis of TCGA datasets, we observed an overexpression of *PRDM7* gene in hepatocarcinoma (LIHC) specimens [45]. Further, it was potentially associated with the risk of developing cancer in Li-Fraumeni-like syndrome without *TP53* mutations [147].

### 2.8. PRDM8

A whole-exome sequencing analysis on a small group of pituitary adenomas revealed genetic variants in several genes, including *PRDM8*. In the same study, PRDM8 mRNA expression level was approximately five-fold lower in invasive pituitary adenomas specimens compared with non-invasive ones [148]. Otherwise, this gene was hypomethylated in primary and metastatic endometrial cancers [149]. Recently, in two independent cohorts of hepatocellular carcinoma patients, a PRDM8 downregulation related to a shorter recurrence-free survival was observed [150]. Both in vitro and in vivo experiments demonstrated that PRDM8 exerts its antitumor role by suppressing the PI3K/AKT/mTOR signaling cascade through the regulation of nucleosome assembly protein 1-like 1 (NAP1L1) (Figure 3B) [150]. Suitably, our TCGA analysis found PRDM8 among the most downregulated *PRDM* genes across human cancers thus suggesting a general role as a tumor suppressor gene [45]. Moreover, our mutation profiling and OncodriveCLUST analysis indicated that it could be a driver gene in pancreas adenocarcinoma, where it is frequently mutated (16% of cases) [45].

### 2.9. PRDM9

PRDM9 is an essential enzyme in the progression of early meiotic prophase playing a key role in the mechanisms of homologous recombination in most mammals [151]. Indeed, it influences the genetic exchange by determining the locations of meiotic recombination hotspots, where genetic recombination occurs. Particularly, through its zinc fingers it binds DNA at specific sites in the genome, where it trimethylates histone H3 at lysine 4 and 36 at surrounding nucleosomes [6]. These recognition sites are 13-mer motifs, which are enriched in human hotspots. Furthermore, PRDM9 interacts with other proteins to form complexes that facilitate the association of hotspots with the chromosomal axis and affect the subsequent programmed DSB initiation and repair, thus allowing genetic exchange between chromosomes (Figure 4). Of note, in the absence of PRDM9, these DNA breaks cannot be repaired properly leading to several human pathologies, such as male infertility [151]. Indeed, meiotic recombination is crucial for accurate chromosomal disjunction and genomic stability maintenance during meiosis; likewise, homologous recombination also promotes genomic stability by repairing DSBs in cells undergoing mitosis. Furthermore, the two processes involve overlapping molecular machinery and comparable mechanisms whose dysregulation can often lead to diseases, including cancer. Accordingly, PRDM9 was suggested to also interfere with mitotic genome regulation [152]. A recent study designed to map human PRDM9 binding sites revealed that human PRDM9 frequently bound regulatory elements of protein coding genes, despite their low recombination rates, and it was able to activate the expression of a subset of genes including the spermatogenesis-specific *CTCFL* and *VCX* genes, thus providing evidence for novel functions of this protein [153].

The first meta-analysis of clinical data sets aimed to identify new cancer/testis genes and investigate the expression profiles of human meiotic genes in normal and cancer tissues and cell lines; several highly specific cancer biomarker genes, including *PRDM9*, were recognized as genes with oncogenic characteristics [154].

Later, sequencing data revealed a substantial excess of rare allelic forms of *PRDM9* in a cohort of parents with children affected from B-cell precursor acute lymphoblastic leukemia (B-ALL). This association was successfully replicated in an independent cohort of children with B-ALL, where this excess was found particularly in aneuploid and infant B-ALL patients [155]. Since PRDM9 variability has been suggested to influence genomic instability, the authors of this study argued that these rare allelic forms could be involved in the development of preleukemic clones in B-ALL patients and proposed that an altered PRDM9 function in the parental germline could lead to the genomic instability associated with childhood ALL [155]. Strikingly, these findings were then confirmed in additional independent populations [156,157].

*PRDM9* mutations have also been correlated with specific solid tumors. Indeed, in a study defining a landscape of non-coding RNA (ncRNA) in the head and neck squamous cell carcinoma (HNSCC), 307 non-coding transcripts differentially expressed in HNSCC were significantly correlated with patient survival, and associated with known gene mutations and chromosome alterations, including *PRDM9* mutations; particularly, *piR-34736* was upregulated two-fold in HNSCC and correlated to patient survival and *PRDM9* mutation [158]. Very recently, a mutation analysis of histone lysine methyltransferases in bladder cancer from TCGA datasets identified *PRDM9* among the six genes with a potential critical role in oncogenesis and prognosis of this cancer type [159]. Noteworthy, our pan-cancer mutation analysis recognized *PRDM9* as one of the most mutated genes of the PRDM family with frequencies ranging from 0.5% to 15.4% and higher than 5% in multiple cancers, such as DLBCL, HNSCC, endometrial, esophageal, stomach, and colon carcinomas, kidney and lung tumors, and melanoma [45].

In a newly published pan-cancer analysis of TCGA data the authors revealed that aberrant expression of PRDM9 was associated with an enrichment of somatic structural variants at sites of binding and activity in several cancer types, thus hypothesizing a novel mechanism underlying genomic instability during tumorigenesis based on the possibility that there are putative uncharacterized genomic features and binding sites leading to these variants [160]. Taken together these pan-cancer studies strongly indicate a role of PRDM9 in oncogenesis possibly through its function in DSB repair.

### 2.10. PRDM10

Using RNA-seq and other methodologies, analysis of 84 soft tissue sarcomas revealed that a significant subset of low-grade undifferentiated pleomorphic sarcoma (UPS) showed a gene fusion of *PRDM10* either with *MED12* or *CITED2*, suggesting that these rearrangements were specific for this less aggressive UPS subset [161]. Moreover, *PRDM10*-rearranged soft tissue tumors are characterized by pleomorphism and a low mitotic count [162]. Currently studies using integrated bioinformatics and network analysis have also recognized the role of PRDM10 in the onset, progression, and drug resistance of many malignancies, such as hepatocellular, prostate, and nasopharyngeal carcinoma, as well as gastric and rectum cancers [163,164,165,166,167,168]; although in our previous TGCA analysis, *PRDM10* was either mutated or overexpressed in certain cancer types [45]. As a possible mechanism, PRDM10 protein could affect *BCL2* gene expression at the transcription level, thus influencing apoptosis (Figure 2) [169].

### 2.11. PRDM11

In childhood ALL, whole-exome-sequencing and whole-genome-sequencing revealed that homozygous non-synonymous coding mutations negatively affected PRDM11 function [170]. In addition, PRDM11 showed anti-tumorigenic effects in an Eμ-Myc mouse model of B cell lymphoma. Indeed, deletion of *PRDM11* accelerated Myc-driven lymphomagenesis, while its overexpression induced apoptosis and delayed lymphoma onset. Moreover, patients with *PRDM11*-deficient DLBCL, belonging to the nongerminal center B-cell-like subtype, had poorer overall survival. A putative mechanism involved the direct expression regulation of key oncogenes such as Fos and Jun (Figure 3G) [171].

It is well known that pseudogenes regulate the expression of protein-coding genes through their function as microRNA sponges. In a recent study, an integrative systems biology approach was applied to identify disease pseudogenes based on a ceRNA (competitive endogenous RNA) hypothesis in lung adenocarcinoma (LUAD). Interestingly, PRDM11 was recognized as part of a triple ceRNA (miR-21-5p-NKAPP1-PRDM11) associated with the poor prognosis of LUAD [172].

### 2.12. PRDM12

Several studies indicated that *PRDM12* might act as a tumor suppressor gene in human chronic myeloid leukemia with derivative chromosome 9 deletions or rearrangements [173,174,175]. Moreover, integrated analysis of genetic abnormalities of the KMT in PC from TCGA, identified *PRDM12* as a gene with a pathogenetic role in this type of cancer [176]. Our previous pan-cancer analysis on TCGA data showed that PRDM12 is upregulated in many tumors including colon cancer [45] and interestingly, is not expressed in adult normal tissues. Accordingly, another study also found that this gene is expressed only in dorsal root ganglia but not in other adult tissues, thus explaining its established role in pain perception [177]. Therefore, further studies investigating the involvement of PRDM12 in malignancies and its possible use as a tumor biomarker are warranted.

### 2.13. PRDM13

PRDM13 was reported for the first time as a tumor suppressor in medulloblastoma, in a study aimed at identifying novel tumor markers and targets for brain tumor immunotherapy through the isolation of tumor antigens by the SEREX (serological analysis of cDNA expression libraries) approach [178]. A more recent analysis included *PRDM13* among genes with differentially methylated CpG sites in men with PC recurrence compared to men with no evidence of recurrence, with higher methylation levels observed in the men with recurrent PC [179].

An in vitro study demonstrated that PRDM13 was involved in malignant glioma cell progression since the overexpression of PRDM13 was able to inhibit proliferation, migration, and invasion of U87 cells and decreased the percentage of cells in the S-phase of the cell cycle, suggesting that PRDM13 may influence DNA replication. PRDM13 inhibited cell proliferation by upregulating *INCA1*, a CDK inhibitor and *ADAMTS12*, a novel antitumor protease that modulates the extracellular signal-regulated kinase signaling pathway (Figure 3G). Additionally, PRDM13 upregulated deleted in liver cancer 1 (*DLC1*) and Rho GTPase-activating protein 30 (*ARHGAP30*) genes, thus inhibiting cell invasion (Figure 1D). The main limitation of this in vitro study was the usage of only one cell line [180]. Thus, future investigations on the possible involvement of PRDM13 in glioma are required, taking into account the analysis of further cell lines and tissue specimens. These results could provide the biological basis for a novel therapeutic approach in glioma. Interestingly, in our pan-cancer reanalysis of the RNA-sequencing datasets from TCGA, we observed a high overexpression of PRDM13 in many cancer types; these included carcinomas from head and neck, bladder, kidney, lung, cervical, and colorectal tissues [45]. Furthermore, exome data indicated that this gene is generally mutated at low frequencies in tumors, with lung squamous cell carcinoma (LUSC) as the most mutated one (3.4%) [45].

### 2.14. PRDM14

In the last decade, several studies have ascribed an oncogenic role to PRDM14. In a panel of breast cancer cell lines and tissue specimens, PRDM14 was highly expressed at both mRNA and protein levels whereas low or no expression was observed in non-cancerous tissues. The analysis of gene copy number suggested that the mechanism could be gene amplification on chromosome 8q13, a region frequently altered in a wide variety of human tumors [181]. Of note, *PRDM14* gene amplification was associated with high mitotic index, histological grade, and distant metastasis in breast cancer specimens [182,183,184]; although no significant differences in copy number were observed between ductal carcinoma in situ and adjacent infiltrating ductal cancer [183]. In vitro experiments showed that while ectopic PRDM14 expression enhanced cancer cell growth and resistance to chemotherapeutic drugs, PRDM14 knockdown was able to induce apoptosis and increase sensitivity to chemotherapeutic drugs [181]. It is well known that PRDM14 is specifically expressed in preimplantation embryos, primordial germ cells, and embryonic stem cells, where it ensures pluripotency by either repressing or activating its target genes via multiple epigenetic mechanisms [185]. More recently, in vitro and in vivo experiments were set up to ascertain whether and how PRDM14 could also confer stem cell-like properties and epigenetic changes to cancer cells. As expected, PRDM14 resulted as markedly expressed in cancer tissues and correlated with poor survival of breast cancer patients. More importantly, PRDM14 was required for the stemness phenotypes of breast cancer cells and induced epigenetic changes finally regulating the expression of genes involved in cancer stemness, metastasis, and chemoresistance. Specifically, it reduced DNA methylation of proto-oncogene and stemness gene promoters, whereas it enhanced methylation of tumor suppressor genes in cancer cells [186]. PRDM14 silencing strongly reduced the stem cell phenotype and inhibited breast cancer cell line proliferation, tumorsphere formation, and suppressed cell growth in the presence of low concentrations of anticancer drugs. Furthermore, the effects of PRDM14 specific siRNAs were also investigated in vivo on the growth of tumor xenografts and metastasis with successful results and no adverse effects; these beneficial effects were also confirmed in a conditional *Prdm14* knockout MMTV-Wnt-1 transgenic mice, a spontaneous murine model of breast cancer suggesting that PRDM14 inhibition may be an effective and novel therapy targeting cancer stem cells (Figure 5) [186,187].

Interestingly, recent in vitro and in vivo experiments established that hyperglycemia could contribute to the worsening of breast malignancies by promoting invasion and hyperactivation of the cancer stem cells; mechanistically, miR-424→cdc42→prdm14 axis could be the key molecular signaling cascade influencing breast cancer progression in diabetic patients [188]. In particular, miR-424 knockdown induces cdc42 expression that in turn positively regulates PRDM14 through the activation of p-21-activated kinase 1 (pak1) and stat5 (Figure 1F). Moreover, in triple negative cancer cells PRDM14 directly binds the heat shock proteins HSP90α and glucose-regulated protein 78 (GRP78) through the C-terminal region containing the zinc finger motifs. Since these genes were overexpressed in several cancers, including triple negative breast cancers, and their inhibitors were able to counteract some cancer phenotypes, these interactions might provide useful targets for cancer treatment [189].

Since the first observation, PRDM14 has been implicated in many other cancer types, most of them showing a similar mechanism involving its role in stem cell pluripotency. For instance, a mouse lymphoblastic leukemia (LL) with retroviral integration displayed aberrant expression of Prdm14 [190]. In addition, PRDM14 was upregulated in about 25% of human lymphoid neoplasms with increased frequencies in T-cell ALL and hyper-diploid precursor B-cell ALL. Interestingly, through the use of mice transduced with bone marrow cells carrying a Prdm14-expressing vector and microarray analysis, it was demonstrated that this gene is able to initiate leukemia due to expansion of a cell population with features of common lymphoid progenitors, which expressed high levels of genes involved in pluripotency, tumor initiation, early B-lineage commitment, the oncogenic Wnt/Ras signaling pathways, and EMT [191]. As in human ALL, mice with LL induced by Prdm14 aberrant expression showed widespread aneuploidy and copy number alterations, indicating significant chromosomal damage due to failure to activate genes involved in chromosomal stability and DNA repair pathways [192]. The inducible overexpression of Prdm14 in a mouse model prompted a rapid-onset and highly penetrant T-cell ALL with high levels of activated NOTCH1 and its downstream targets [193]. More recently, in a derived inducible FLAG-PRDM14 mouse model, PRDM14-induced T-cell ALL was driven by activated Notch1 through a RAG-mediated deletion-based mechanism (Figure 3E and Figure 5) [194]. Furthermore, novel findings demonstrated that PRDM14 requires the master hematopoietic regulator CBFA2T3 to initiate leukemia in progenitor cells [195].

In the last years, PRDM14 has been widely studied in lung cancer. Indeed, immunohistochemistry and Western blotting followed by univariate and multivariate analyses were used to examine PRDM14 expression in both primary lung cancers and matched lymph node metastases and to determine the association between its expression and prognosis. As result, increased PRDM14 expression was found to significantly correlate with differentiation of radically resected tumors and was an independently significant predictor of patient survival. These findings suggested that this gene could be a potential biomarker for predicting unfavorable prognosis in non-small cell lung cancer [196]. Consistently, PRDM14 overexpression promoted cell migration through extracellular matrix degradation in a human lung cancer cell line [197]. Otherwise, PRDM14 inhibited 293T cell proliferation by influencing G1/S-phase transition and impacted cell migration by regulating the expression of matrix metalloproteinase (MMP)/tissue inhibitor of metalloproteinases (TIMP) suggesting a cell context behavior (Figure 1F) [198]. A multiplex ligation-dependent probe amplification study in tumors and matched normal tissues from 82 patients with non-small cell lung cancer, found frequent copy number alterations of chromosome 8, with *PRDM14* as the most frequently amplified gene [199]. More recently, overexpression of PRDM14 was observed also in pancreatic cancer, where it could sustain cell pluripotency; indeed, PRDM14 knockdown suppressed cancer stem-like phenotypes, including liver metastasis, via miR-125a-3p regulating Fyn expression (Figure 1F) [200]. Additionally, PRDM14-positive cells were also detected through immunohistochemistry analyses in precursor pancreatic intraepithelial neoplasia and chronic pancreatitis lesions, a pancreatic cancer risk factor [201]. Alterations of the 8q13.2 region with *PRDM14* copy number gain were also found in intracranial germ cell tumors [202] and in head and neck cancer [203].

A genome-wide association studies (GWAS) analysis identified nine new susceptibility loci associated with testicular germ cell tumors; these loci comprise genes conceivably related to the development of this cancer, including *PRDM14,* which is essential for early germ cell specification [204]. Accordingly, elevated PRDM14 expression was recently detected in all the tested germ cell tumors, a heterogeneous group of tumors occurring in gonadal and extragonadal locations, except for teratomas; in these tumors PRDM14 may act by blocking differentiation [205].

Overall, the oncogenic functions of PRDM14 described so far involve its full-length isoform containing the PR domain; however, it is unknown whether a *PR*− isoform exists for PRDM14, or whether it has tumor suppressor activity. Indeed, possible behavior as a tumor suppressor gene has emerged from several studies reporting alterations in the *PRDM14* promoter methylation. Analysis of high-risk human papillomavirus (HPV)-positive cervical scrapes revealed significantly increased methylation of three genes, including *PRDM14*, in women with high-grade cervical disease compared to controls [206]. Later, *PRDM14* silencing was found through hypermethylation of its promoter in HPV-positive cancers, and ectopic expression of PRDM14 in HPV16-positive cancer cell lines induced apoptosis, possibly due to a direct upregulation of the pro-apoptotic genes *NOXA* and *PUMA* [207]. Although this different role of PRDM14 could be explained with the expression of HPV oncogenic proteins in those tumors, the same epigenetic changes were found also in other cancer types. Particularly, significant increases in *PRDM14* methylation were revealed in high-grade non-muscle invasive bladder cancer and in colon carcinogenesis [208,209]. Similarly, *PRDM14* was recently added to a gene panel utilized, also in clinical trials, for the diagnosis of lung cancer through the analysis of DNA hypermethylation of biomarkers in sputum, thus denoting its silencing in these tumors [210,211,212]. Collectively, these findings indicate a role for *PRDM14* as a tumor suppressor gene; this would be in contrast with the above described studies, which reported a general overexpression of this gene in several tumors, including lung cancer, suggestive of an oncogenic activity [196,197]. In order to elucidate this dual role of PRDM14 in cancer further studies are still required. In particular, the potential existence of alternative isoforms with different activities should be investigated; moreover, proteomic and biochemical analyses in pan-cancer could add novel insights to this topic.

### 2.15. PRDM15

*PRDM15* was identified as a candidate tumor suppressor gene, since localized homozygous deletions were revealed at 21q22 chromosome region in several pancreatic cancer cell lines [213]. Then, microarray-based studies recognized *PRDM15* as a novel overexpressed gene in human B cell lymphomas [214]. More recently, recurrent mutations were observed in refractory patients with DLBCL [215]. Of note, a recent mechanistic study demonstrated that PRDM15 modulates the transcription of upstream regulators of Wnt and MAPK-ERK signaling to safeguard naive pluripotency [216]. Particularly, PRDM15 regulates *Spry1* and *Rspo1* transcription through the direct binding of a specific DNA sequence in their promoter regions. Activation of *Rspo1* and *Spry1* gene expression leads to inhibition of the MAK-ERK pathway and activation of Wnt/β-catenin pathway, respectively (Figure 5).

### 2.16. PRDM16

*PRDM16* gene was initially designated as *MEL1* (*MDS1/EVI1*-like gene 1), because it shared the same domain structure as *MDS1/EVI1* (*MECOM*); it was expressed in leukemia cells with t(1;3)(p36;q21) but not in other cell lines or tissues, suggesting that this gene could be specifically activated in a subset of myelodysplastic syndrome and AML [217]. Of note, two translation products were identified, the full-length protein MEL1 (now PRDM16 or fPRDM16) and an alternative short product, MEL1S (sPRDM16), mostly lacking the amino-terminal PR domain and expressed mainly in t(1;3)(p36;q21)-positive leukemia cells. Since MEL1S overexpression blocked induced granulocytic differentiation of murine myeloid cell lines, this isoform was considered as one of the possible causative factors in the pathogenesis of myeloid leukemia [218]. Moreover, the aberrant expression of MEL1S/sPRDM16, associated with DNA hypomethylation, was correlated with dysregulation of TGF-β-mediated signaling suggesting that MEL1S might be responsible for TGF-β resistance in leukemogenesis of adult T-cell leukemia [219]. Noteworthy, its overexpression was also shown to induce myeloid leukemia in p53 knock-outmice through an abnormal growth of stem and progenitor cells [220]. Then, *PRDM16* was also found as the fusion partner of *RUNX1* in the t(1;21)(p36;q22), a recurrent chromosome abnormality associated with therapy-related AML [221,222]. Other data suggested that the *RUNX1/PRDM16* fusion gene could contribute to immortalization of the leukemic stem cell and play an important role during clonal evolution from chronic myeloid leukemia (CML) to AML and imatinib resistance [223,224]. In addition, cryptic and partial deletions of *PRDM16* and RUNX1 without t(1;21)(p36;q22) and/or *RUNX1-PRDM16* fusion were detected in a case of progressive CML suggesting that different mechanisms of chromosomal rearrangement may occur in these malignancies [225]. Successively, additional *PRDM16* translocation partners, fusion transcripts and other rearrangements have been detected in leukemia cases with a poor prognosis, most of them showing an upregulation of this gene as a common feature [226,227,228,229,230,231].

Actually, several studies have established that high PRDM16 expression is independently associated with adverse outcomes in both adult and pediatric AML patients [232,233,234,235]. Accordingly, low expression of PRDM16/MEL1 was associated with favorable disease and suggested as a specific anti-leukemic mechanism [236,237]. More recently, a study suggested that different mechanisms, acting at protein level, like SUMOylation of sPRDM16, might also play an important role in the progression of AML [238]. Interestingly, *PRDM16* displays various similarities with *EVI1/MECOM*. For instance, *PRDM16* is also a common target site for viral integration [239,240,241]. Moreover, these *PRDM* genes both codify for short and full-length protein isoforms and are mutually involved in AML rearrangements [232,242,243]. The mouse model of conditional Prdm16 deletion elucidated the role of these two isoforms in normal and leukemic hematopoiesis and identified sPrdm16 as one of the drivers of prognostically adverse inflammation in leukemia [244]. Notably, unlike the *PR*− isoforms, both full-length isoforms of PRDM16 and MECOM exhibited a significantly enriched association with components of the NuRD chromatin remodeling complex [245].

To date, rearrangements of the chromosomal region encompassing *PRDM16* have been observed not only in hematopoietic malignancies but also in several solid tumors though with different and/or conflicting results, which altogether indicate this gene may function as both oncogene and tumor suppressor gene. For instance, genome-wide array based comparative genomic hybridization (array-CGH) defined distinct amplifications in osteosarcoma, involving *PRDM16* [246]. Otherwise, array-CGH integrated with gene expression analysis of leiomyosarcoma revealed a frequent loss at 1p36, which contains *PRDM16,* suggesting that this defect could promote muscle differentiation in this context [247]. Similarly, integrated analysis of uterine leiomyosarcoma revealed *PRDM16* deletions and/or reduced expression [248]. In DNA array-CGH analysis of normal and sporadic colorectal cancer specimens, analyses of gains of focal minimal common regions also included *PRDM16* among the candidate oncogenes of these malignancies [249]. Moreover, droplet digital PCR analysis to detect copy number variations identified recurrent gains at *PRDM16* region in hemangioblastoma pathogenesis [250]. Previously, *MEL1/PRDM16*, together with *SKI*, was aberrantly expressed by chromosomal co-amplification of 1p36.32 in gastric cancer cells [251]. Knockdown of these two genes synergistically restored TGF-β responsiveness in gastric cancer cells and reduced tumor growth in vivo; biochemical analysis demonstrated that MEL1/PRDM16 interacted with SKI and inhibited TGF-β signaling by stabilizing the inactive Smad3-SKI complex on the promoter of TGF-β target genes (Figure 3D) [251]. Similarly, *PRDM16* high copy gain was also observed in a small cohort using CGH [252]. Otherwise, a more recent study showed that miR-214 was able to inhibit PRDM16 expression thus promoting the proliferation and migration of gastric cancer cells and enhancing the Warburg effect (Figure 3H) [253].

A possible role as a tumor suppressor gene has been proposed in lung cancer where PRDM16 is aberrantly methylated and its expression is low or absent [139,140]. Accordingly, the median overall survival of both non-small cell lung cancer and LUAD patients with high levels of PRDM16 was significantly longer than that of cases with low levels of this gene [254,255,256]. Both in vitro and in vivo analyses established that PRDM16 was capable of inhibiting the EMT of cancer cells by repressing the transcription of Mucin-4 (*MUC4*), one of the regulators of EMT in lung adenocarcinomas (Figure 1E) [256]. Otherwise, PRDM16 was overexpressed in astrocytoma patients due to promoter hypomethylation and this high expression level correlated with poor prognosis [257]. The same authors proved that the tumor suppressor miR-101 could reverse the *PRDM16* hypomethylation status thus suppressing its expression through direct epigenetic regulation, finally leading to mitochondrial disruption and apoptosis [257]. Similarly, *PRDM16* hypomethylation was also described in glioblastoma [258] whereas a study suggested that *PRDM16* hypermethylation level might be used as a potential biomarker for the diagnosis of esophageal cancer [259]. Altogether, these findings indicate that *PRDM16* methylation status, both hypermethylation and hypomethylation, is often affected in distinct cancers, where this gene can play alternatively a role as an oncogene or as a tumor suppressor gene.

Weighted gene expression network analysis has defined *PRDM16* among the hub genes in renal cell carcinoma with an effect on patient survival suggesting it might be considered as a novel candidate biomarker of this malignancy [260]. Additionally, PRDM16 is highly overexpressed also in atypical teratoid/rhabdoid tumor, a highly malignant brain tumor predominantly arising in infants; moreover, it could have a functional role in human rhabdoid tumor cells since PRDM16 knockdown resulted in reduced metabolic activity and proliferation [261]. A different approach identified PRDM16 as a possible therapeutic target in PC. This study aimed to elucidate the function of human HMTs and histone demethylase genes through RNA interference screening; PRDM16 was associated with evasion from apoptosis, and its spliced form, sPRDM16, was found to be aberrantly expressed in PC cells [262].

It is accepted that PRDM16 plays a crucial role in the determination and function of brown and beige fat [12]. Although previous evidence implicated white adipocytes in promoting aggressive breast tumor behavior, a few studies have also associated brown adipocytes with this disease. Specifically, expression of beige and brown adipose markers from host and tumor cells influenced breast tumor growth in nude mice suggesting that manipulation of browning could represent a novel strategy to inhibit tumor development [263]. Notably, browning of white adipose tissue is one of the significant factors contributing to energy wasting in cancer cachexia. Two recent studies have also elucidated the underlying molecular mechanism in mice. Firstly, the tumor secretory factor ZAG stimulated PPARγ and early B cell factor 2 expression and promoted their recruitment to the Prdm16 promoter, leading to enhanced expression of this PRDM member, which in turn determined brown cell fate [264]. Then, a recent evaluation found that exosomes derived from gastric cancer cells could deliver the circular RNA ciRS-133 into preadipocytes, promoting the differentiation of preadipocytes into brown-like cells through PRDM16 activation and miR-133 suppression. Moreover, knockdown of ciRS-133 reduced cancer cachexia in tumor-implanted mice, decreasing oxygen consumption and heat production. These findings suggest that exosome-delivered circRNAs are involved in adipose browning and play a key role in cancer-associated cachexia by targeting the miR-133/PRDM16 pathway [265].

Our TCGA analysis found that *PRDM16* gene was mutated in about 6–7% of DLBCL and it was frequently altered in many cases of skin cutaneous melanoma (7.8%) and endometrial carcinoma (5.6%) [45]. Furthermore, a *PRDM16* downregulation was observed in most of the analyzed malignancies [45].

### 2.17. ZNF408/PRDM17

This gene is associated with familial retinitis pigmentosa and vitreoretinopathy [266]. To date, no associations have been found with cancer; likewise, our TCGA analysis showed a slight overexpression of this gene in some tumors and a low percentage of mutations across cancers, with the highest frequency at 4% [45].

### 2.18. ZFPM1/FOG1

*ZFPM1* was firstly named as friend of GATA1 (FOG) due to its isolation through the yeast two-hybrid system using the conserved zinc finger DNA-binding domain of GATA1 [267]. Its protein product bound GATA1 and acted as a transcriptional cofactor of this essential hematopoietic regulator. It is co-expressed with GATA1 during embryonic development and hematopoiesis. Furthermore, FOG and GATA1 synergistically activate transcription from a hematopoietic-specific regulatory region and cooperate during both erythroid and megakaryocytic cell differentiation [267]. Later, an additional GATA1 independent FOG function also in the early stage of megakaryocyte development was suggested [268].

As expected from its function during hematopoiesis, several studies have shown a deregulation of ZFPM1 and its related pathway in hematologic malignancies. For instance, an upregulation of genes involved in erythroid differentiation, including *GATA1* and *ZFPM1/FOG1*, was revealed in molecular high-risk patients with cytogenetically normal AML [269]. Accordingly, tumor necrosis factor (TNF)-alpha treatment inhibited differentiation in erythroleukemia induced cell lines through a decreased FOG1 expression and a consequent decreased level of GATA1/FOG1 complex [270]. Likewise, wogonin, a flavonoid highly effective to treat hematologic malignancies, was able to induce erythroid differentiation and cell cycle arrest in CML cells via regulating the function of GATA1 and FOG1. Morever, wogonin significantly prolonged survival of CML-bearing mice by cell proliferation inhibition suggesting that GATA1/FOG1 complex could be a target for CML treatment [271].

Furthermore, a study revealed that the leukemogenic CBFβ-SMMHC (CM) fusion protein, which is known to disrupt myeloid and lymphoid differentiation, was able to affect adult erythropoiesis and create stress-resistant preleukemic progenitors predisposed to malignant transformation; of note, these progenitors displayed dysregulated erythroid and megakaryocytic fate determining factors including reduced ZFPM1/FOG1 [272]. Additional mechanistic results from forced FOG1 expression in human K562 erythroleukemia cells indicated that FOG1 has an important role in inducing cells to differentiate toward the erythroid lineage rather than the myelo-lymphoid one by repressing the expression of PU.1 [273].

Further insights came from a more recent study investigating the effect of immunomodulatory drugs (IMiDs), analogues of thalidomide, on megakaryopoiesis. Despite their high response rates in multiple myeloma patients, these drugs are also associated with severe thrombocytopenia; however, the induction mechanism is still unknown. Results from this study showed that IMiDs could induce self-renewal and proliferation of megakaryocytic progenitors by downregulating *GATA1, ZFPM1/FOG1,* and *NFE2* expression, finally leading to inhibition of megakaryocyte maturation [274].

Interestingly, more recent literature has suggested a possible involvement of ZFPM1 in solid tumors. Indeed, in a first report *ZFPM1* was identified as a new susceptibility locus for testicular germ cell tumors in a multistage GWAS with a combined data set of >25,000 individuals [275]. Moreover, high expression of ZFPM1 was related with good prognosis of LUAD through the analysis of RNA-seq, DNA methylation, and miRNA-seq data of squamous cell cancer samples downloaded from TCGA [276]. Noteworthy, in the last months, two studies analyzing mutation data from TCGA with different approaches have both assigned a role as a driver gene in human adrenocortical carcinoma (ACC). Indeed, our pan-cancer analysis revealed *ZFPM1/FOG1* mutation occurrence in about 50% of ACC patients. Interestingly, these mutations were localized in a hotspot region and OncodriveCLUST results suggested it could be putatively considered a cancer driver gene in this malignancy [45]. These results were confirmed by a complete analysis of the mutational data of ACC, which was performed on the same public database and validated through further algorithms (Mutsig and 20/20 rule); this study identified a new ACC-specific gene mutation signature, also comprising *ZFPM1* among the six genes [277]. In addition, in our analysis we found that *ZFPM1* is mutated also in colorectal cancers at relatively high frequencies [45].

### 2.19. ZFPM2/FOG2

A second cofactor for GATA family members, designated FOG-related factor (FOG2), codified by *ZFPM2*, was identified in independent studies; it is particularly abundant in both murine and human hearts and brains where it plays a key role during development [278,279]. FOG2 is able to physically interact with all six known GATA family members (mainly with GATA4/5/6) by binding to their N-terminal zinc finger [278,279]. Interaction of FOG2 with GATA4 leads to modulation of GATA4 transcriptional activity that can be either stimulatory or repressive, depending on the specific promoter and the cell type [278,279].

Regarding its role in cancer, an initial observation established that FOG2, GATA4, and GATA6 were expressed in human ovary and cultured ovarian somatic cells; moreover, their abundant expression was detected in the human sex cord-derived ovarian tumors (granulosa and theca cell tumors) suggesting they could also play a role in the growth and differentiation of ovarian stromal cells [279]. Later, animal model studies defined that the GATA4-FOG2 complex and FOG2 were both required for embryonic ovarian development until the sex determination stage whereas postnatal regulation depended only on GATA4 [280]. Furthermore, the altered expression of these factors also in ovarian granulosa cell tumors and in pediatric sex cord-stromal tumors of the ovary, suggested that they may play a role in tumor growth and progression of these cancers and may represent a prognostic factor in some sex cord-derived tumors [281,282]. Similarly, GATA4, GATA6, and FOG2 are known to function during fetal testis development and are all expressed in mouse and human Sertoli and Leydig cells; of note, they are present also in cells from carcinoma in situ testis, which is the pre-invasive precursor of testicular germ cell cancer, the most common malignancy among young males [283].

It is known that GATA3 is an essential regulator of mammary-gland morphogenesis and luminal-cell differentiation; FOG2 was shown to act during mammary development even though the consequences of *Gata3* gene ablation in murine models were more severe than that of *Fog2*, whose excision induced premature mammary gland involution [284]. Accordingly, while a consolidated role has been established for GATA3 in human breast carcinogenesis, only few indirect observations suggest a protective role for ZFPM2/FOG2 in this tumor [284,285,286].

In the last decade, several disease-association studies and RNA and protein expression analyses have revealed a potential role for this gene, mainly as a tumor suppressor, in other cancer types. Most of these studies explored *ZFPM2/FOG2* together with the genes encoding for GATA factors. For instance, immunohistochemistry, real-time RT-PCR, and microarray analysis showed a downregulation of ZFPM2/FOG2 in aggressive but not in favorable primary neuroblastoma [287]. Bioinformatics analysis of microarray data and genotyping of a *ZFPM2* polymorphism indicated its possible role in glioma and glioblastoma [288,289]. Similarly, integrated network analysis of microarray data predicted *ZFPM2* as one of the key genes regulated by parathyroid hormone receptor 1 in osteosarcoma [290]. Moreover, *ZFPM2-ELF5* was identified by RNA sequencing among the fusion gene transcripts in multicystic mesothelioma [291], whereas a ZFPM2 microdeletion was found in a case of adult Wilms tumor [292]. More recently, a study reported the discovery of a novel lncRNA, ZFPM2 antisense RNA 1 (ZFPM2-AS1) and its critical role in gastric carcinogenesis through p53 pathway attenuation [293]. Interestingly, ZFPM2-AS1 has been also included in a seven-lncRNA signature for prognosis prediction in hepatocellular carcinoma [294].

Finally, an interesting finding from a GWAS meta-analysis reported both *ZFPM1* and *ZFPM2* as genes influencing the circulating levels of the angiogenic vascular endothelial growth factor (VEGF), which is known to impact various physiological and disease processes including cancer [295].

Altogether, the results from the above-mentioned studies only provide minor independent clues for a tumor suppressor role of this gene. A significant insight could derive from our TCGA analysis [45]. Indeed, we found that, after *PRDM9*, *ZFPM2/FOG2* was the most mutated PRDM gene with pan-cancer frequencies of protein-affecting mutations higher than 1%; specifically, we detected mutation frequencies higher than 5% in patients with skin cutaneous melanoma, lung tumors, uterine carcinosarcoma, esophageal carcinoma, and stomach and rectum adenocarcinomas [45]. Noteworthy, this gene was also the most downregulated gene among the members of this family [45].

## 3. Clinical Value of PRDMs in Cancer and Concluding Remarks

The framework outlined by the current knowledge on the PRDM protein family functions, strongly suggests a valuable potential in cancer management, including diagnosis, prognosis, and therapy. Indeed, the emerging data from TCGA analysis and other studies highlight tumor-specific alterations of *PRDM* genes in all the tumors analyzed, where their expression was also significantly changed compared to normal tissues (Table 1) [45]. The relevance of tumor-specificity lies in the possibility of a novel tool for diagnosis or prognosis, not only relative to an organ-specific tumor but also to its different subtypes. This evidence implies and puts emphasis on the possible use of PRDMs as novel targets of therapeutic intervention that, in the last years, have become even more urgent.

In this scenario, being involved in a multitude of pathways regulating several cancer-related processes, ranging from cell metabolism to stemness, the pharmacological control of PRDM epigenetic modulators represent a potential multi-target approach in cancer therapy (Figure 5). A hallmark of cancer is the genomic instability that accounts for the increase in mutation frequency. It is well known that genomic integrity is closely monitored by several surveillance mechanisms, including a DNA damage checkpoint, DNA repair machinery, and mitotic checkpoint. A defect in the regulation of any of these mechanisms often results in genomic instability, which predisposes the cell to malignant transformation. Interestingly, PRDM2/RIZ1 and PRDM9 are implied in the DSB repair complex, which is essential for ensuring accurate DNA repair and maintenance of genomic integrity [85] (Figure 4).

Cell transformation is a multi-step process characterized by an increased proliferation rate and survival, invasiveness, and metastasis. Some PRDMs are strictly implied in the regulation of such processes. Indeed, PRDM proteins mediate the transduction of a plethora of cell signals like steroid hormones, TGF-β, or epidermal growth factor (EGF) and control the expression of growth factors, like insulin-like growth factor-1 (IGF-1) that modulate cell behavior (Figure 3). For instance, PRDM2a/RIZ1 was shown to counteract the IGF-1 receptor and the downstream components ERK1/2 and AKT (Figure 3B) [73]. Additionally, it is well known that upon ligand binding, the transcription factor ERα, a ligand-regulated transcription factor, stimulates proliferative and anti-apoptotic signaling pathways in target tissues, such as human breast cancer cells. PRDM2a/RIZ1 is a downstream effector of estrogen action and is related to estrogen-regulated cancer cell proliferation [76]. Indeed, ERα modulates the PRDM2/RIZ isoforms intracellular concentration ratio, by an indirect and selective decrease of RIZ1 expression and a transcriptional activation of RIZ2 (Figure 3C) [49]. Moreover, MEL1/PRDM16 and MECOM/PRDM3 both exert a negative regulation of TGF-β signaling, an important player in cytostasis and normal epithelium differentiation that is altered in several malignancies. Specifically, MEL1/PRDM16 interplays by stabilizing the inactive Smad3-SKI complex on the promoter of TGF-β target genes whereas MECOM/PRDM3 binds and inactivates SMAD3 proteins (Figure 3D) [115,117,251]. Similarly, several evidences revealed that PRDMs might act also by regulating tyrosine kinase receptor (RTK) signaling. PRDM3, through its first zinc finger domain, associates and inhibits JNK activity, thus protecting cells from stress-induced cell death that is dependent on JNK activation (Figure 3F) [120]. On the contrary, PRDM5 upregulates JNK expression (Figure 3F) [143]. Additionally, PRDM11 represses the oncogenes Fos and Jun that are frequently induced by aberrant growth factor signaling or oncogenic activation of MAP kinase cascade, such as constitutively active RAS (Figure 3G) [171].

Apoptosis is a mechanism useful to eliminate unnecessary or damaged cells, whose alteration is frequently observed in cancers. Although the precise involvement of PRDMs is not unraveled in this process, they are able to control the expression of different genes, such as *BCL2* and *TP53* genes, which play a pivotal role in apoptosis (Figure 2). For instance, PRDM1/BLIMP1 binds to the *TP53* promoter and represses its transcription. p53, in turn, binds to and positively regulates PRDM1/*BLIMP1* [45]. Furthermore, PRDM10 and MECOM/PRDM3 induce the expression of the gene encoding the anti-apoptotic factor Bcl-2 while PRDM14 directly regulates the pro-apoptotic genes *NOXA* and *PUMA* [119,169,207]. Furthermore, PRDM5 overexpression causes apoptosis of cancer cells (Figure 2) [131].

Furthermore, in healthy cells and early-stage cancer cells, TGF-β signaling exerts a tumor-suppressor function, including cell-cycle arrest and apoptosis. However, its activation in late-stage cancer can contribute to metastasis and chemoresistance by inducing EMT. The EMT is involved in spreading, stemness, and drug-resistance of cancer cells and represents a feature observed in more aggressive and metastatic tumors. Particularly, TGF-β1 promotes the expression of the transcriptional repressor PRDM1/BLIMP1 via c-Raf (RAF1) and AP-1 pathway. PRDM1/BLIMP1, in turn, by reducing the expression of *BMP-5*, induces the expression of Snail (SNAI1), the EMT master regulator (Figure 1B) [38]. Other PRDMs have been directly implicated in EMT (Figure 1). For example, PRDM2 controls the expression of genes involved in EMT, with vimentin being the most significantly regulated gene (Figure 1E) [61]. PRDM16, on the contrary, inhibits EMT by repressing the transcription of MUC4 (Figure 1E) [256]. Moreover, PRDM14 promotes cell migration by regulating the expression level of MMP/TIMP, genes involved in EMT [198]. PRDM14 knockdown decreased cancer stem-like phenotypes through upregulation of miR-125a-3p that subsequently downregulated Fyna mechanism that is reported to regulate tumor phenotypes in pancreatic cancer (Figure 1F) [200].

Important insights arise from the role of some PRDMs in the control of Wnt/β-catenin and Notch pathways. It is extensively reported that Wnt and Notch signaling are widely associated with chemoresistance and cancer stem cells control, in different tumors, including colon, breast, pancreatic cancers, and gliomas [296]. In recent years, the need of an effective strategy to eradicate cancer stem cells, considering the tumor fuel, has become evident. Interestingly, PRDM1 and PRDM5 antagonize the Wnt/β-catenin pathway. PRDM1 reduces the expression of Dkk1 while PRDM5 forms a chromatin complex with CBP, TCF, and β-catenin that prevent the expression of Wnt target genes (Figure 3A and Figure 5) [43,137]. Similarly, PRDM15 modulates the transcription of upstream Wnt regulators [216]. Of note, through direct regulation of Notch pathway, PRDM14 was certainly associated with cancer stem cells and thus chemoresistance [186]. Indeed, its silencing in breast cancer cells reduces cancer stem cells phenotype and tumorsphere formation (Table 1 and Figure 1). In addition, in T-cell ALL, PRDM14 binds an intron of *Notch1* gene and modifies the chromatin structure by inducing H3K4me3, allowing the access of the RAG recombinase complex. RAG deletes part of the *Notch1* promoter and consequently a truncated, ligand independent Notch1 protein is produced (Figure 3E and Figure 5) [193,194].

Alterations in cellular metabolism are among the most consistent hallmarks of cancer and the variability of metabolic reprogramming among cancer cells emerges from the relative contributions of different oncogenes and tumor-suppressor genes. Cancer cells rewrite their metabolism to support a rapid proliferation, survival, invasion, and metastasis. The enzyme monoacylglycerol lipase (MAGL) was shown to supply and to orchestrate a fatty acid network enriched in oncogenic signaling lipids that promote tumor progression and in vivo tumor growth [297]. MAGL blockade impairs migration, invasiveness, and tumorigenicity in aggressive human cancer [297]. *MAGL* is a Wnt responsive gene upregulated by Prdm5 loss and Wnt activation and Prdm5 was found able to target and regulate MAGL in mouse fibroblasts and the small intestine [134]. Intriguingly, PRDM5 protein expression is downregulated in human colon neoplastic lesions [134]. Beyond the well-recognized role exerted by PRDM16 in the differentiation and function of brown and beige fat, the increase in the Warburg effect arising from the downregulation of *PRDM16* [251] strongly enforces PRDMs as players in the metabolic reprogramming of tumor growth. Particularly, hypoxia-induced miR-214 inhibits PRDM16 expression, thus promoting both cell proliferation and migration and enhancing the Warburg effect (Figure 3H) [253].

Additionally, since some PRDMs (e.g., PRDM1, 9, 11, 14, 16) are able to orchestrate the differentiation of B and T lymphocytes, their involvement in the control of a more incisive immune response to neoplasia cannot be excluded.

Finally, the recent discovery of MRK-740 as a potent, selective and cell-active substrate-competitive PRDM9 inhibitor, opens the way for targeting the PRDM subfamily of SAM-dependent methyltransferases [298].

Overall, this evidence dictates the interest in the PRDM protein family. Nevertheless, an important issue to consider is the existence of PRDM protein isoforms with distinct and opposing functions, as oncosuppressors or oncogenes. However, this dual behavior has been explored only for a small number of members even though it is expected to be a general feature of this family [1,2,3]. Currently, as reported for other families, many studies have highlighted the consequences of aberrant isoform expression in triggering tumorigenesis and drug resistance, suggesting that dysregulation of alternative transcription may be a key mechanism leading to cancer progression and drug resistance [299]. Thus, it is evident that future studies will be moved to a complete and deep characterization of each member of the PRDM family, in order to optimize their use as diagnostic or prognostic tools and improve pharmacological research.

## Figures and Tables

**Table 1:** Cancer specific alterations of PRDM family members.

Gene Symbol (Previous Symbols/Synonyms)	Cancer Type	Molecular Alteration	Putative Effect/Mechanism	References
*PRDM1* *(PRDI-BF1; BLIMP1)*	Lymphoma (Diffuse large B cell lymphoma, extranodal NK (natural killer)/T-cell lymphoma)	Inactivating mutations, chromosomal deletion, and epigenetic silencing	Putative tumor suppressor gene. It is downregulated or silenced in human DLBCL (diffuse large B cell lymphoma) and other haematological malignancies. The activation of B cell lymphoma (Bcl)-2/Ras pathway stimulates RelB and p130Cas/ErbB2 invasion leading to its overexpression	[13,14,15,16,17,18,19,20,21,22,23,24,25,26,27,28,29,30,31,32,33,34,35,36,37,38,39,40,41,42,43,44,45,46]
	Breast cancer	Upregulated		
	Lung cancer	Downregulated		
	Glioma	Downregulated		
*PRDM2* *(RIZ; RIZ1; RIZ2; KMT8; MTB-ZF; HUMHOXY1; KMT8A)*	Neuroblastoma, hepatoma, colorectal, ovarian, and breast cancers, chronic myelocytic leukemia, non-Hodgkin’s lymphoma, melanoma, parathyroid adenoma, Merkel cell carcinoma, and pheochromocytoma	Aberrant isoform expression Up/downregulated	The imbalance of its main protein isoforms, Riz1 and Riz2, (through promoter DNA methylation, frameshift, and missense mutations) may constitute an important cause of malignancy with the *PR*+ plus product commonly lost or downregulated and the *PR*− isoform always present at higher levels in cancer cells. It modulates estrogen receptor signaling in breast cancer	[3,45,47,48,49,50,51,52,53,54,55,56,57,58,59,60,61,62,63,64,65,66,67,68,69,70,71,72,73,74,75,76,77,78,79,80,81,82,83,84,85]
	Colorectal, gastric, endometrial, pancreatic, Microsatellite instability positive cancers	Frameshift mutations		
	Prostate, endometrial cancer	Polymorphisms		
	Breast carcinomas, liver tumors, colon and lung cancer	Methylation		
*MECOM* *(MDS1-EVI1; PRDM3; KMT8E)*	Acute myeloid leukemia	Chromosomal rearrangements or proviral insertion	Tumor suppressor gene: short PR- isoform (EVI1) is overexpressed or rearranged in cancer	[7,45,86,87,88,89,90,91,92,93,94,95,96,97,98,99,100,101,102,103,104,105,106,107,108,109,110,111,112,113,114,115,116,117,118,119,120,121,122,123,124,125,126,127]
	Ovarian cancer	Downregulated		
	Colon cancer	Integrated bioinformatics and network analyses		
	Colorectal cancer	Frameshift mutation		
*PRDM4* *(PFM1)*	Ovarian, gastric, and pancreatic cancer	Deletion	Maps to frequently deleted locus (12q23-q24.1). It could contribute to YAP (yes-associated protein)-induced tumorigenesis possibly via mediating the expression of other YAP target genes, which finally contribute to cell invasion and metastasis promotion	[128,129,130]
	Gastric cancer	Upregulated		
*PRDM5* *(PFM2)*	Breast and ovarian cancer, cervical carcinoma, liver carcinoma, gastric and colorectal cancer, lung cancer, nasopharyngeal and esophageal carcinoma	Silenced	Silenced in several human cancers through aberrant DNA methylation. Ectopic overexpression induced G2/M arrest and apoptosis in cancer cell lines. Its tumor suppressor function could be explained at least in part through negative regulation of aberrant Wnt/β-catenin signaling and oncogene expression	[131,132,133,134,135,136,137,138,139,140,141,142,143]
*PRDM6* *(PRISM; KMT8C)*	Bladder cancer	Downregulated	Transcriptional repressor involved in the regulation of endothelial cell proliferation, survival and differentiation	[45,144,145,146]
	Breast cancer	Susceptibility gene variants		
*PRDM7* *(ZNF910)*	Hepatocellular carcinoma	Upregulated	Potentially associated with the risk of developing cancer in a Li-Fraumeni-like syndrome patients without TP53 mutations	[45,147]
*PRDM8* *(KMT8D)*	Pituitary adenomas	Downregulated	Its alterations are mostly associated with metastasis. Mechanistically, it suppresses the PI3K/AKT/mTOR signaling cascade through the regulation of nucleosome assembly protein 1-like 1. It could be a driver gene in pancreas adenocarcinoma	[45,148,149,150]
	Endometrial cancer	Hypomethylated		
	Hepatocellular carcinoma	Downregulated		
	Pancreas adenocarcinoma	Frequently mutated		
*PRDM9* *(MSBP3; PFM6; ZNF899; KMT8B)*	Acute lymphoblastic leukemia and diffuse large B cell lymphoma	Frequent mutations and rare allelic variants	Key role in the mechanisms of homologous recombination. Indeed, it facilitates the association of hotspots with the chromosomal axis and affects the subsequent programmed DNA double-strand breaks initiation and repair. Rare allelic variants were associated with acute lymphoblastic leukemia	[6,45,151,152,153,154,155,156,157,158,159,160]
	Head and neck squamous cell carcinoma, endometrial, esophageal, stomach and colon carcinomas, kidney and lung tumors and melanoma			
*PRDM10* *(KIAA1231; PFM7; MGC131802)*	Soft tissue sarcoma	Gene fusions	Gene fusions were found in many cases of low-grade undifferentiated pleomorphic sarcoma. It could influence apoptosis by affecting Bcl-2 expression	[45,161,162,163,164,165,166,167,168,169]
	Hepatocellular carcinoma, nasopharyngeal carcinoma, gastric cancer, rectum cancer	Integrated bioinformatics and network analyses		
*PRDM11* *(PFM8)*	Diffuse large B cell lymphoma	Non-synonymous coding mutations	Its deletion accelerated Myc-driven lymphomagenesis whereas overexpression induced apoptosis and delayed lymphoma onset in a mouse model. Part of a ceRNA (competitive endogenous RNA) triple (miR-21-5p-NKAPP1-PRDM11) associated with the poor prognosis of lung adenocarcinoma	[170,171,172]
	Lung adenocarcinoma	Integrative systems biology approach		
*PRDM12* *(PFM9)*	Chronic myeloid leukemia	Chromosome rearrangements	Chromosome rearrangements in chronic myeloid leukemia. Its overexpression showed anti-proliferative properties in vitro	[45,173,174,175,176,177]
	Prostate cancer, colon cancer	Upregulated		
*PRDM13* *(PFM10)*	Medulloblastoma	Immunotherapy target	Its overexpression was able to inhibit proliferation, migration, and invasion of malignant glioma cells.	[45,178,179,180]
	Prostate cancer	Hypermethylated		
	Head and neck squamous cell carcinoma, bladder, kidney, lung, cervical, and colorectal cancers	Upregulated		
*PRDM14*	Lymphoblastic leukemia	Upregulated	Its aberrant high expression observed in human lymphoid malignancies, breast cancer, and other neoplasms may be ascribed to either gene amplification on chromosome 8q13 or copy number gain. Functionally, its requirement in the stemness phenotypes could also explain the involvement in the proliferation and migration of cancer cells. However, a dual role, as both oncogene and tumor suppressor gene, has been recently described in several human cancers and needs to be investigated	[181,182,183,184,185,186,187,188,189,190,191,192,193,194,195,196,197,198,199,200,201,202,203,204,205,206,207,208,209,210,211,212]
	Breast cancer	Gene amplification/copy number gain		
	Lung cancer, head and neck cancer, germ cell tumors			
	Cervical, bladder, colon, and lung cancers	Promoter methylation		
*PRDM15* *(ZNF298; C21orf83)*	Pancreatic cancer	Homozygous deletions	It modulates the transcription of upstream regulators of Wnt and MAPK-ERK signaling to safeguard naive pluripotency	[213,214,215,216]
	Diffuse large B cell lymphoma	Recurrent mutations		
*PRDM16* *(MEL1; PFM13; KIAA1675; MGC166915; KMT8F)*	Myeloid leukemia	Aberrant isoform expression/gene fusion/mutations	As for other *PRDM* genes, two main products were identified, with the short PR-l isoform (sPRDM16) displaying oncogenic properties; indeed, this variant could induce myeloid leukemia in p53 knock-out KO mice and was responsible for transforming growth factor (TGF)-β resistance in leukemogenesis. *PRDM16* gene fusions with *RUNX1* and other partners could also contribute to these hematological malignancies. Further genetic and epigenetic alterations have been observed in brain and other solid tumors, where also the short isoform may function. Recently, a role in cancer cachexia has been suggested owing to its function in adipose browning	[45,217,218,219,220,221,222,223,224,225,226,227,228,229,230,231,232,233,234,235,236,237,238,239,240,241,242,243,244,245,246,247,248,249,250,251,252,253,254,255,256,257,258,259,260,261,262,263,264,265]
	Prostate cancer	Aberrant isoform expression		
	Brain tumors	Upregulated by hypomethylation		
	Osteosarcoma, colon cancer, renal cell carcinoma	Gene amplification		
	Leiomyosarcoma, gastric, lung, and esophageal cancer	Gene deletion/reduced expression		
	Skin melanoma, endometrial carcinoma	Frequently mutated		
*ZNF408* *(PRDM17; FLJ12827)*	-	-	No associations have been found with cancer	[58,266]
*ZFPM1* *(FOG1; FOG; ZNF89A; ZC2HC11A)*	Acute myeloid leukemia, chronic myeloid leukemia	Upregulated	Forced FOG1 (friend of GATA-1) expression in human erythroleukemia cells suggested an important role in inducing differentiation toward the erythroid lineage rather than the myelo-lymphoid one by repressing the expression of PU.1. Putative cancer driver gene in adrenocortical carcinoma since recurrent mutations (50%) with a hotspot region were found in this neoplasm. Frequent mutations were also observed in colon and rectum adenocarcinomas	[45,267,268,269,270,271,272,273,274,275,276,277]
	Adrenocortical carcinoma, colon and rectum adenocarcinomas	Frequently mutated		
	Testicular germ cell tumors	Genome wide association studies		
	Lung adenocarcinoma	Upregulated by hypomethylation		
*ZFPM2* *(FOG2; hFOG-2; ZNF89B; ZC2HC11B)*	Ovarian tumors	Upregulated	Putative function as tumor suppressor gene. Mostly, it is downregulated and frequently mutated in many cancer types	[45,278,279,280,281,282,283,284,285,286,287,288,289,290,291,292,293,294,295]
	Neuroblastoma	Downregulated		
	Mesothelioma	Fusion gene		
	Skin cutaneous melanoma, lung cancers uterine carcinosarcoma, esophageal carcinoma, stomach and rectum adenocarcinoma	Frequently mutated		

## Abbreviations

ABC	Activated-B cell like
ACC	Adrenocortical carcinoma
ALL	Acute lymphoblastic leukemia
AML	Acute myeloid leukemia
ARHGAP30	Rho GTPase-activating protein 30
ATM	Ataxia telangiectasia mutated
CEBPβ	CCAAT/enhancer-binding protein beta-2 isoform
ceRNA	Competitive endogenous RNA
CIN	Chromosomal Instability
CML	Chronic myeloid leukemia
DKK	Dickkopf-1 LDL low-density lipoprotein
DLBCL	Diffuse large B cell lymphoma
DLC	Deleted in liver cancer
DSB	Double-strand break
EGF	Epidermal growth factor
EMT	Epithelial-to-mesenchymal transition
ER	Estrogen receptor
FOG	Friend of GATA
GCB	Germinal center B-cell
GRP78	Glucose-regulated protein 78
HDAC	Histone deacetylases
HMT	Histone methyltransferases
HNSCC	Head and neck squamous cell carcinoma
HPV	Human papillomavirus
IGF-1	Insulin-like growth factor-1
IMiDs	Immunomodulatory drugs
ITGB2	Leukocyte-specific integrin β2
KMT	Lysine methyltransferases
LL	Lymphoblastic leukemia
LUAD	Lung adenocarcinoma
MAGL	Monoacylglycerol lipase
MIN	Microsatellite Instability
MMP	Matrix Metalloproteinase
MUC4	Mucin-4
NAP1L1	Nucleosome assembly protein 1-like 1
PC	Prostate cancer
PRDM	PRD-BF1 and RIZ homology domain containing
RTK	Tyrosine kinase receptor
SERPIN	Serine protease inhibitor
TCGA	The Cancer Genome Atlas
TGF	Transforming growth factor
TIMP	Tissue inhibitor of metalloproteinases
TXNIP	Thioredoxin binding protein
VEGF	Vascular endothelial growth factor
YAP	Yes-associated protein

## References

[1] Di Zazzo E., De Rosa C., Abbondanza C., Moncharmont B. (2013). PRDM Proteins: Molecular Mechanisms in Signal Transduction and Transcriptional Regulation. Biology.

[2] Mzoughi S., Tan Y.X., Low D., Guccione E. (2016). The role of PRDMs in cancer: One family, two sides. Curr. Opin. Genet. Dev..

[3] Sorrentino A., Rienzo M., Ciccodicola A., Casamassimi A., Abbondanza C. (2018). Human PRDM2: Structure, function and pathophysiology. Biochim. Biophys. Acta Gene Regul. Mech..

[4] Clifton M.K., Westman B.J., Thong S.Y., O’Connell M.R., Webster M.W., Shepherd N.E., Quinlan K.G., Crossley M., Blobel G.A., Mackay J.P. (2014). The identification and structure of an N-terminal PR domain show that FOG1 is a member of the PRDM family of proteins. PLoS ONE.

[5] Hayashi K., Yoshida K., Matsui Y. (2005). A histone H3 methyltransferase controls epigenetic events required for meiotic prophase. Nature.

[6] Eram M.S., Bustos S.P., Lima-Fernandes E., Siarheyeva A., Senisterra G., Hajian T., Chau I., Duan S., Wu H., Dombrovski L. (2014). Trimethylation of histone H3 lysine 36 by human methyltransferase PRDM9 protein. J. Biol. Chem..

[7] Pinheiro I., Margueron R., Shukeir N., Eisold M., Fritzsch C., Richter F.M., Mittler G., Genoud C., Goyama S., Kurokawa M. (2012). Prdm3 and Prdm16 are H3K9me1 methyltransferases required for mammalian heterochromatin integrity. Cell.

[8] Huang S., Shao G., Liu L. (1998). The PR domain of the Rb-binding zinc finger protein RIZ1 is a protein binding interface and is related to the SET domain functioning in chromatin-mediated gene expression. J. Biol. Chem..

[9] Ren B., Chee K.J., Kim T.H., Maniatis T. (1999). PRDI-BF1/Blimp-1 repression is mediated by corepressors of the Groucho family of proteins. Genes Dev..

[10] Seale P., Bjork B., Yang W., Kajimura S., Chin S., Kuang S., Scimè A., Devarakonda S., Conroe H.M., Erdjument-Bromage H. (2008). PRDM16 controls a brown fat/skeletal muscle switch. Nature.

[11] Okashita N., Suwa Y., Nishimura O., Sakashita N., Kadota M., Nagamatsu G., Kawaguchi M., Kashida H., Nakajima A., Tachibana M. (2016). PRDM14 Drives OCT3/4 Recruitment via Active Demethylation in the Transition from Primed to Naive Pluripotency. Stem Cell Rep..

[12] Chi J., Cohen P. (2016). The Multifaceted Roles of PRDM16: Adipose Biology and Beyond. Trends Endocrinol. Metab..

[13] Keller A.D., Maniatis T. (1991). Identification and characterization of a novel repressor of beta-interferon gene expression. Genes Dev..

[14] Turner C.A., Mack D.H., Davis M.M. (1994). Blimp-1, a novel zinc finger-containing protein that can drive the maturation of B lymphocytes into immunoglobulin-secreting cells. Cell.

[15] Martins G.A., Cimmino L., Shapiro-Shelef M., Szabolcs M., Herron A., Magnusdottir E., Calame K. (2006). Transcriptional repressor Blimp-1 regulates T cell homeostasis and function. Nat. Immunol..

[16] Kallies A., Hawkins E.D., Belz G.T., Metcalf D., Hommel M., Corcoran L.M., Hodgkin P.D., Nutt S.L. (2006). Transcriptional repressor Blimp-1 is essential for T cell homeostasis and self-tolerance. Nat. Immunol..

[17] De Mel S., Hue S.S., Jeyasekharan A.D., Chng W.J., Ng S.B. (2019). Molecular pathogenic pathways in extranodal NK/T cell lymphoma. J. Hematol. Oncol..

[18] Boi M., Zucca E., Inghirami G., Bertoni F. (2015). PRDM1/BLIMP1: A tumor suppressor gene in B and T cell lymphomas. Leuk. Lymphoma.

[19] Pasqualucci L., Compagno M., Houldsworth J., Monti S., Grunn A., Nandula S.V., Aster J.C., Murty V.V., Shipp M.A., Dalla-Favera R. (2006). Inactivation of the PRDM1/BLIMP1 gene in diffuse large B cell lymphoma. J. Exp. Med..

[20] Tam W., Gomez M., Chadburn A., Lee J.W., Chan W.C., Knowles D.M. (2006). Mutational analysis of PRDM1 indicates a tumor-suppressor role in diffuse large B-cell lymphomas. Blood.

[21] Iqbal J., Kucuk C., Deleeuw R.J., Srivastava G., Tam W., Geng H., Klinkebiel D., Christman J.K., Patel K., Cao K. (2009). Genomic analyses reveal global functional alterations that promote tumor growth and novel tumor suppressor genes in natural killer-cell malignancies. Leukemia.

[22] Küçük C., Iqbal J., Hu X., Gaulard P., De Leval L., Srivastava G., Au W.Y., McKeithan T.W., Chan W.C. (2011). PRDM1 is a tumor suppressor gene in natural killer cell malignancies. Proc. Natl. Acad. Sci. USA.

[23] Boi M., Rinaldi A., Kwee I., Bonetti P., Todaro M., Tabbò F., Piva R., Rancoita P.M., Matolcsy A., Timar B. (2013). PRDM1/BLIMP1 is commonly inactivated in anaplastic large T-cell lymphoma. Blood.

[24] Nie K., Gomez M., Landgraf P., Garcia J.F., Liu Y., Tan L.H., Chadburn A., Tuschl T., Knowles D.M., Tam W. (2008). MicroRNA-mediated down-regulation of PRDM1/Blimp-1 in Hodgkin/Reed-Sternberg cells: A potential pathogenetic lesion in Hodgkin lymphomas. Am. J. Pathol..

[25] Nie K., Zhang T., Allawi H., Gomez M., Liu Y., Chadburn A., Wang Y.L., Knowles D.M., Tam W. (2010). Epigenetic down-regulation of the tumor suppressor gene PRDM1/Blimp-1 in diffuse large B cell lymphomas: A potential role of the microRNA let-7. Am. J. Pathol..

[26] Xia Y., Xu-Monette Z.Y., Tzankov A., Li X., Manyam G.C., Murty V., Bhagat G., Zhang S., Pasqualucci L., Visco C. (2017). Loss of PRDM1/BLIMP-1 function contributes to poor prognosis of activated B-cell-like diffuse large B-cell lymphoma. Leukemia.

[27] Mandelbaum J., Bhagat G., Tang H., Mo T., Brahmachary M., Shen Q., Chadburn A., Rajewsky K., Tarakhovsky A., Pasqualucci L. (2010). BLIMP1 is a tumor suppressor gene frequently disrupted in activated B cell-like diffuse large B cell lymphoma. Cancer Cell.

[28] Wan Z., Lu Y., Rui L., Yu X., Li Z. (2016). PRDM1 overexpression induce G0/G1 arrest in DF-1 cell line. Gene.

[29] Calado D.P., Zhang B., Srinivasan L., Sasaki Y., Seagal J., Unitt C., Rodig S., Kutok J., Tarakhovsky A., Schmidt-Supprian M. (2010). Constitutive canonical NF-κB activation cooperates with disruption of BLIMP1 in the pathogenesis of activated B cell-like diffuse large cell lymphoma. Cancer Cell.

[30] Liu J., Liang L., Li D., Nong L., Zheng Y., Huang S., Zhang B., Li T. (2019). JAK3/STAT3 oncogenic pathway and PRDM1 expression stratify clinicopathologic features of extranodal NK/T-cell lymphoma, nasal type. Oncol. Rep..

[31] Baytak E., Gong Q., Akman B., Yuan H., Chan W.C., Küçük C. (2017). Whole transcriptome analysis reveals dysregulated oncogenic lncRNAs in natural killer/T-cell lymphoma and establishes MIR155HG as a target of PRDM1. Tumour Biol..

[32] Zhang Z., Liang L., Li D., Nong L., Liu J., Qu L., Qu L., Zheng Y., Zhang B., Li T. (2017). Hypermethylation of PRDM1/Blimp-1 promoter in extranodal NK/T-cell lymphoma, nasal type: An evidence of predominant role in its downregulation. Hematol. Oncol..

[33] Liang L., Zhang Z., Wang Y., Nong L., Zheng Y., Qu L., Zhang B., Li T. (2015). The Genetic Deletion of 6q21 and PRDM1 and Clinical Implications in Extranodal NK/T Cell Lymphoma, Nasal Type. Biomed. Res. Int..

[34] Yu J., Angelin-Duclos C., Greenwood J., Liao J., Calame K. (2000). Transcriptional repression by blimp-1 (PRDI-BF1) involves recruitment of histone deacetylase. Mol. Cell Biol..

[35] Gyory I., Wu J., Fejér G., Seto E., Wright K.L. (2004). PRDI-BF1 recruits the histone H3 methyltransferase G9a in transcriptional silencing. Nat. Immunol..

[36] Montes-Moreno S., Martinez-Magunacelaya N., Zecchini-Barrese T., Villambrosía S.G., Linares E., Ranchal T., Rodriguez-Pinilla M., Batlle A., Cereceda-Company L., Revert-Arce J.B. (2017). Plasmablastic lymphoma phenotype is determined by genetic alterations in MYC and PRDM1. Mod. Pathol..

[37] Wang X., Belguise K., O’Neill C.F., Sánchez-Morgan N., Romagnoli M., Eddy S.F., Mineva N.D., Yu Z., Min C., Trinkaus-Randall V. (2009). RelB NF-kappaB represses estrogen receptor alpha expression via induction of the zinc finger protein Blimp1. Mol. Cell Biol..

[38] Romagnoli M., Belguise K., Yu Z., Wang X., Landesman-Bollag E., Seldin D.C., Chalbos D., Barillé-Nion S., Jézéquel P., Seldin M.L. (2012). Epithelial-to-mesenchymal transition induced by TGF-β1 is mediated by Blimp-1-dependent repression of BMP-5. Cancer Res..

[39] Sciortino M., Camacho-Leal M.D.P., Orso F., Grassi E., Costamagna A., Provero P., Tam W., Turco E., Defilippi P., Taverna D. (2017). Dysregulation of Blimp1 transcriptional repressor unleashes p130Cas/ErbB2 breast cancer invasion. Sci. Rep..

[40] Yan J., Jiang J., Lim C.A., Wu Q., Ng H.H., Chin K.C. (2007). BLIMP1 regulates cell growth through repression of p53 transcription. Proc. Natl. Acad. Sci. USA.

[41] Györy I., Fejér G., Ghosh N., Seto E., Wright K.L. (2003). Identification of a functionally impaired positive regulatory domain I binding factor 1 transcription repressor in myeloma cell lines. J. Immunol..

[42] Liu C., Banister C.E., Weige C.C., Altomare D., Richardson J.H., Contreras C.M., Buckhaults P.J. (2018). PRDM1 silences stem cell-related genes and inhibits proliferation of human colon tumor organoids. Proc. Natl. Acad. Sci. USA.

[43] Wang X., Wang K., Han L., Zhang A., Shi Z., Zhang K., Zhang H., Yang S., Pu P., Shen C. (2013). PRDM1 is directly targeted by miR-30a-5p and modulates the Wnt/β-catenin pathway in a Dkk1-dependent manner during glioma growth. Cancer Lett..

[44] Zhu Z., Wang H., Wei Y., Meng F., Liu Z., Zhang Z. (2017). Downregulation of PRDM1 promotes cellular invasion and lung cancer metastasis. Tumour Biol..

[45] Sorrentino A., Federico A., Rienzo M., Gazzerro P., Bifulco M., Ciccodicola A., Casamassimi A., Abbondanza C. (2018). PR/SET Domain Family and Cancer: Novel Insights from the Cancer Genome Atlas. Int. J. Mol. Sci..

[46] Desmots F., Roussel M., Pangault C., Llamas-Gutierrez F., Pastoret C., Guiheneuf E., Le Priol J., Camara-Clayette V., Caron G., Henry C. (2019). Pan-HDAC Inhibitors Restore PRDM1 Response to IL21 in CREBBP-Mutated Follicular Lymphoma. Clin. Cancer Res..

[47] Liu L., Shao G., Steele-Perkins G., Huang S. (1997). The retinoblastoma interacting zinc finger gene RIZ produces a PR domain-lacking product through an internal promoter. J. Biol. Chem..

[48] Huang S. (1999). The retinoblastoma protein-interacting zinc finger gene RIZ in 1p36-linked cancers. Front. Biosci..

[49] Abbondanza C., De Rosa C., D’Arcangelo A., Pacifico M., Spizuoco C., Piluso G., Di Zazzo E., Gazzerro P., Medici N., Moncharmont B. (2012). Identification of a functional estrogen-responsive enhancer element in the promoter 2 of PRDM2 gene in breast cancer cell lines. J. Cell. Physiol..

[50] Jiang G.L., Huang S. (2000). The yin–yang of PR-domain family genes in tumorigenesis. Histol. Histopathol..

[51] Kim K.C., Geng L., Huang S. (2003). Inactivation of a histone methyltransferase by mutations in human cancers. Cancer Res..

[52] He L., Yu J.X., Liu L., Buyse I.M., Wang M.S., Yang Q.C., Nakagawara A., Brodeur G.M., Shi Y.E., Huang S. (1998). RIZ1, but not the alternative RIZ2 product of the same gene, is underexpressed in breast cancer, and forced RIZ1 expression causes G2-M cell cycle arrest and/or apoptosis. Cancer Res..

[53] Rossi M., Abbondanza C., D’Arcangelo A., Gazzerro P., Medici N., Moncharmont B., Puca G.A. (2004). The Zn-finger domain of RIZ protein promotes MCF-7 cell proliferation. Cancer Lett..

[54] Chambery A., Farina A., Di Maro A., Rossi M., Abbondanza C., Moncharmont B., Malorni L., Cacace G., Pocsfalvi G., Malorni A. (2004). Proteomic analysis of MCF-7 cell lines expressing the zinc-finger or the proline-rich domain of retinoblastoma-interacting-zincfinger protein. J. Proteome Res..

[55] Steele-Perkins G., Fang W., Yang X.H., Van Gele M., Carling T., Gu J., Buyse I.M., Fletcher J.A., Liu J., Bronson R. (2001). Tumor formation and inactivation of RIZ1, an Rb-binding member of a nuclear protein methyltransferase superfamily. Genes Dev..

[56] Piao Z., Fang W., Malkhosyan S., Kim H., Horii A., Perucho M., Huang S. (2000). Frequent frameshift mutations of RIZ in sporadic gastrointestinal and endometrial carcinomas with microsatellite instability. Cancer Res..

[57] Jiang G.L., Huang S. (2001). Adenovirus expressing RIZ1 in tumor suppressor gene therapy of microsatellite-unstable colorectal cancers. Cancer Res..

[58] Sakurada K., Furukawa T., Kato Y., Kayama T., Huang S., Horii A. (2001). RIZ, the retinoblastoma protein interacting zinc finger gene, is mutated in genetically unstable cancers of the pancreas, stomach, and colorectum. Genes Chromosomes Cancer.

[59] Chadwick R.B., Jiang G.L., Bennington G.A., Yuan B., Johnson C.K., Stevens M.W., Niemann T.H., Peltomaki P., Huang S., de la Chapelle A. (2000). Candidate tumor suppressor RIZ is frequently involved in colorectal carcinogenesis. Proc. Natl. Acad. Sci. USA.

[60] Maruvka Y.E., Mouw K.W., Karlic R., Parasuraman P., Kamburov A.P., Haradhvala N.J., Hess J.M., Rheinbay E., Brody Y., Koren A. (2017). Analysis of somatic microsatellite indels identifies driver events in human tumors. Nat. Biotechnol..

[61] Pandzic T., Rendo V., Lim J., Larsson C., Larsson J., Stoimenov I., Kundu S., Ali M.A., Hellström M., He L. (2017). Somatic PRDM2 c.4467delA mutations in colorectal cancers control histone methylation and tumor growth. Oncotarget.

[62] Poetsch M., Dittberner T., Woenckhaus C. (2002). Frameshift mutations of RIZ, but no point mutations in RIZ1 exons in malignant melanomas with deletions in 1p36. Oncogene.

[63] Sasaki O., Meguro K., Tohmiya Y., Funato T., Shibahara S., Sasaki T. (2002). Nucleotide alteration of retinoblastoma protein-interacting zinc finger gene, RIZ, in human leukemia. Tohoku J. Exp. Med..

[64] Fang W., Piao Z., Simon D., Sheu J.C., Huang S. (2000). Mapping of a minimal deleted region in human hepatocellular carcinoma to 1p36.13-p36.23 and mutational analysis of the RIZ (PRDM2) gene localized to the region. Genes Chromosomes Cancer.

[65] Fang W., Piao Z., Simon D., Sheu J.C., Perucho M., Huang S. (2001). Preferential loss of a polymorphic RIZ allele in human hepatocellular carcinoma. Br. J. Cancer.

[66] Mir R., Najar I.A., Guru S., Javaid J., Yadav P., Masroor M., Zuberi M., Farooq S., Bhat M., Gupta N. (2017). A deletion polymorphism in the RIZ gene is associated with increased progression of imatinib treated chronic myeloid leukemia patients. Leuk. Lymphoma.

[67] Rossi V., Staibano S., Abbondanza C., Pasquali D., De Rosa C., Mascolo M., Bellastella G., Visconti D., De Bellis A., Moncharmont B. (2009). Expression of RIZ1 protein (Retinoblastoma-interacting zinc-finger protein 1) in prostate cancer epithelial cells changes with cancer grade progression and is modulated in vitro by DHT and E2. J. Cell Physiol..

[68] Yang T., Ren C., Jiang A., Yu Z., Li G., Wang G., Zhang Q. (2017). RIZ1 is regulated by estrogen and suppresses tumor progression in endometrial cancer. Biochem. Biophys. Res. Commun..

[69] Du Y., Carling T., Fang W., Piao Z., Sheu J.C., Huang S. (2001). Hypermethylation in human cancers of the RIZ1 tumor suppressor gene, a member of a histone/protein methyltransferase superfamily. Cancer Res..

[70] Zhao Z., Hu Y., Shen X., Lao Y., Zhang L., Qiu X., Hu J., Gong P., Cui H., Lu S. (2017). HBx represses RIZ1 expression by DNA methyltransferase 1 involvement in decreased miR-152 in hepatocellular carcinoma. Oncol. Rep..

[71] Xue Y., Chen R., Du W., Yang F., Wei X. (2017). RIZ1 and histone methylation status in pituitary adenomas. Tumour Biol..

[72] Chang H.W., Chan A., Kwong D.L., Wei W.I., Sham J.S., Yuen A.P. (2003). Detection of hypermethylated RIZ1 gene in primary tumor, mouth, and throat rinsing fluid, nasopharyngeal swab, and peripheral blood of nasopharyngeal carcinoma patient. Clin. Cancer Res..

[73] Pastural E., Takahashi N., Dong W.F., Bainbridge M., Hull A., Pearson D., Huang S., Lowsky R., DeCoteau J.F., Geyer C.R. (2007). RIZ1 repression is associated with insulin-like growth factor-1 signaling activation in chronic myeloid leukemia cell lines. Oncogene.

[74] Medici N., Abbondanza C., Nigro V., Rossi V., Piluso G., Belsito A., Gallo L., Roscigno A., Bontempo P., Puca A.A. (1999). Identification of a DNA binding protein cooperating with estrogen receptor as RIZ (retinoblastoma interacting zinc finger protein). Biochem. Biophys. Res. Commun..

[75] Abbondanza C., Medici N., Nigro V., Rossi V., Gallo L., Piluso G., Belsito A., Roscigno A., Bontempo P., Puca A.A. (2000). The retinoblastoma-interacting zinc-finger protein RIZ is a downstream effector of estrogen action. Proc. Natl. Acad. Sci. USA.

[76] Gazzerro P., Abbondanza C., D’Arcangelo A., Rossi M., Medici N., Moncharmont B., Puca G.A. (2006). Modulation of RIZ gene expression is associated to estradiol control of MCF-7 breast cancer cell proliferation. Exp. Cell Res..

[77] Di Zazzo E., Porcile C., Bartollino S., Moncharmont B. (2016). Critical Function of PRDM2 in the Neoplastic Growth of Testicular Germ Cell Tumors. Biology.

[78] Abbondanza C., De Rosa C., Ombra M.N., Aceto F., Medici N., Altucci L., Moncharmont B., Puca G.A., Porcellini A., Avvedimento E.V. (2011). Highlighting chromosome loops in DNA-picked chromatin (DPC). Epigenetics.

[79] Bhat-Nakshatri P., Wang G., Collins N.R., Thomson M.J., Geistlinger T.R., Carroll J.S., Brown M., Hammond S., Srour E.F., Liu Y. (2009). Estradiol-regulated microRNAs control estradiol response in breast cancer cells. Nucleic Acids Res..

[80] Gazzerro P., Bontempo P., Schiavone E.M., Abbondanza C., Moncharmont B., Ignazio Armetta I., Medici N., De Simone M., Nola E., Puca G.A. (2001). Differentiation of myeloid cell lines correlates with a selective expression of RIZ protein. Mol. Med..

[81] Sun W., Qiao L., Liu Q., Chen L., Ling B., Sammynaiken R., Yang J. (2011). Anticancer activity of the PR domain of tumor suppressor RIZ1. Int. J. Med. Sci..

[82] Ding M.H., Wang Z., Jiang L., Fu H.L., Gao J., Lin X.B., Zhang C.L., Liu Z.Y., Shi Y.F., Qiu G.Z. (2015). The transducible TAT-RIZ1-PR protein exerts histone methyltransferase activity and tumor-suppressive functions in human malignant meningiomas. Biomaterials.

[83] Cai Z., Zou Y., Hu H., Lu C., Sun W., Jiang L., Hu G. (2017). RIZ1 negatively regulates ubiquitin-conjugating enzyme E2C/UbcH10 via targeting c-Myc in meningioma. Am. J. Transl. Res..

[84] Congdon L.M., Sims J.K., Tuzon C.T., Rice J.C. (2014). The PR-Set7 binding domain of Riz1 is required for the H4K20me1-H3K9me1 trans-tail ‘histone code’ and Riz1 tumor suppressor function. Nucleic Acids Res..

[85] Khurana S., Kruhlak M.J., Kim J., Tran A.D., Liu J., Nyswaner K., Shi L., Jailwala P., Sung M.H., Hakim O. (2014). A macrohistone variant links dynamic chromatin compaction to BRCA1-dependent genome maintenance. Cell Rep..

[86] Morishita K., Parker D.S., Mucenski M.L., Jenkins N.A., Copeland N.G., Ihle J.N. (1988). Retroviral activation of a novel gene encoding a zinc finger protein in IL-3-dependent myeloid leukemia cell lines. Cell.

[87] Nucifora G., Laricchia-Robbio L., Senyuk V. (2006). EVI1 and hematopoietic disorders: History and perspectives. Gene.

[88] Fears S., Mathieu C., Zeleznik-Le N., Huang S., Rowley J., Nucifora G. (1996). Intergenic splicing of MDS1 and EVI1 occurs in normal tissues as well as in myeloid leukemia and produces a new member of the PR domain family. Proc. Natl. Acad. Sci. USA.

[89] Wieser R. (2007). The oncogene and developmental regulator EVI1: Expression, biochemical properties, and biological functions. Gene.

[90] Suzukawa K., Parganas E., Gajjar A., Abe T., Takahashi S., Tani K., Asano S., Asou H., Kamada N., Yokota J. (1994). Identification of a breakpoint cluster region 3’ of the ribophorin I gene at 3q21 associated with the transcriptional activation of the EVI1 gene in acute myelogenous leukemias with inv(3)(q21q26). Blood.

[91] Gröschel S., Lugthart S., Schlenk R.F., Valk P.J., Eiwen K., Goudswaard C., van Putten W.J., Kayser S., Verdonck L.F., Lübbert M. (2010). High EVI1 expression predicts outcome in younger adult patients with acute myeloid leukemia and is associated with distinct cytogenetic abnormalities. J. Clin. Oncol..

[92] Lugthart S., van Drunen E., van Norden Y., van Hoven A., Erpelinck C.A., Valk P.J., Beverloo H.B., Löwenberg B., Delwel R. (2008). High EVI1 levels predict adverse outcome in acute myeloid leukemia: Prevalence of EVI1 overexpression and chromosome 3q26 abnormalities underestimated. Blood.

[93] Yuan X., Wang X., Bi K., Jiang G. (2015). The role of EVI-1 in normal hematopoiesis and myeloid malignancies (Review). Int. J. Oncol..

[94] Ayoub E., Wilson M.P., McGrath K.E., Li A.J., Frisch B.J., Palis J., Calvi L.M., Zhang Y., Perkins A.S. (2018). EVI1 overexpression reprograms hematopoiesis via upregulation of Spi1 transcription. Nat. Commun..

[95] Tang Z., Tang G., Hu S., Patel K.P., Yin C.C., Wang W., Lin P., Toruner G.A., Ok C.Y., Gu J. (2019). Deciphering the complexities of MECOM rearrangement-driven chromosomal aberrations. Cancer Genet..

[96] Brooks D.J., Woodward S., Thompson F.H., Dos Santos B., Russell M., Yang J.M., Guan X.Y., Trent J., Alberts D.S., Taetle R. (1996). Expression of the zinc finger gene EVI-1 in ovarian and other cancers. Br. J. Cancer.

[97] Yasui K., Konishi C., Gen Y., Endo M., Dohi O., Tomie A., Kitaichi T., Yamada N., Iwai N., Nishikawa T. (2015). EVI1, a target gene for amplification at 3q26, antagonizes transforming growth factor-β-mediated growth inhibition in hepatocellular carcinoma. Cancer Sci..

[98] Sattler H.P., Lensch R., Rohde V., Zimmer E., Meese E., Bonkhoff H., Retz M., Zwergel T., Bex A., Stoeckle M. (2000). Novel amplification unit at chromosome 3q25-q27 in human prostate cancer. Prostate.

[99] Nanjundan M., Nakayama Y., Cheng K.W., Lahad J., Liu J., Lu K., Kuo W.L., Smith-McCune K., Fishman D., Gray J.W. (2007). Amplification of MDS1/EVI1 and EVI1, located in the 3q26.2 amplicon, is associated with favorable patient prognosis in ovarian cancer. Cancer Res..

[100] Dutta P., Bui T., Bauckman K.A., Keyomarsi K., Mills G.B., Nanjundan M. (2013). EVI1 splice variants modulate functional responses in ovarian cancer cells. Mol. Oncol..

[101] Morishita K., Parganas E., William C.L., Whittaker M.H., Drabkin H., Oval J., Taetle R., Valentine M.B., Ihle J.N. (1992). Activation of EVI1 gene expression in human acute myelogenous leukemias by translocations spanning 300-400 kilobases on chromosome band 3q26. Proc. Natl. Acad. Sci. USA.

[102] Arai S., Yoshimi A., Shimabe M., Ichikawa M., Nakagawa M., Imai Y., Goyama S., Kurokawa M. (2011). Evi-1 is a transcriptional target of mixed-lineage leukemia oncoproteins in hematopoietic stem cells. Blood.

[103] Louz D., van den Broek M., Verbakel S., Vankan Y., van Lom K., Joosten M., Meijer D., Löwenberg B., Delwel R. (2000). Erythroid defects and increased retrovirally-induced tumor formation in Evi1 transgenic mice. Leukemia.

[104] Buonamici S., Li D., Chi Y., Zhao R., Wang X., Brace L., Ni H., Saunthararajah Y., Nucifora G. (2004). EVI1 induces myelodysplastic syndrome in mice. J. Clin. Investig..

[105] Kustikova O.S., Schwarzer A., Stahlhut M., Brugman M.H., Neumann T., Yang M., Li Z., Schambach A., Heinz N., Gerdes S. (2013). Activation of Evi1 inhibits cell cycle progression and differentiation of hematopoietic progenitor cells. Leukemia.

[106] Yoshimi A., Goyama S., Watanabe-Okochi N., Yoshiki Y., Nannya Y., Nitta E., Arai S., Sato T., Shimabe M., Nakagawa M. (2011). Evi1 represses PTEN expression and activates PI3K/AKT/mTOR via interactions with polycomb proteins. Blood.

[107] Glass C., Wuertzer C., Cui X., Bi Y., Davuluri R., Xiao Y.Y., Wilson M., Owens K., Zhang Y., Perkins A. (2013). Global Identification of EVI1 Target Genes in Acute Myeloid Leukemia. PLoS ONE.

[108] Goyama S., Yamamoto G., Shimabe M., Sato T., Ichikawa M., Ogawa S., Chiba S., Kurokawa M. (2008). Evi-1 is a critical regulator for hematopoietic stem cells and transformed leukemic cells. Cell Stem Cell.

[109] Zhang Y., Stehling-Sun S., Lezon-Geyda K., Juneja S.C., Coillard L., Chatterjee G., Wuertzer C.A., Camargo F., Perkins A.S. (2011). PR-domain-containing Mds1-Evi1 is critical for long-term hematopoietic stem cell function. Blood.

[110] Senyuk V., Premanand K., Xu P., Qian Z., Nucifora G. (2011). The oncoprotein EVI1 and the DNA methyltransferase Dnmt3 co-operate in binding and de novo methylation of target DNA. PLoS One..

[111] Pradhan A.K., Halder A., Chakraborty S. (2014). Physical and functional interaction ofthe proto-oncogene EVI1 and tumor suppressor gene HIC1 deregulates Bcl-xLmediated block in apoptosis. Int. J. Biochem. Cell Biol..

[112] Balgobind B.V., Lugthart S., Hollink I.H., Arentsen-Peters S.T., van Wering E.R., de Graaf S.S., Reinhardt D., Creutzig U., Kaspers G.J., de Bont E.S. (2010). EVI1 overexpression in distinct subtypes of pediatric acute myeloid leukemia. Leukemia.

[113] Jazaeri A.A., Ferriss J.S., Bryant J.L., Dalton M.S., Dutta A. (2010). Evaluation of EVI1 and EVI1s (Delta324) as potential therapeutic targets in ovarian cancer. Gynecol. Oncol..

[114] Koos B., Bender S., Witt H., Mertsch S., Felsberg J., Beschorner R., Korshunov A., Riesmeier B., Pfister S., Paulus W. (2011). The transcription factor evi-1 is overexpressed, promotes proliferation, and is prognostically unfavorable in infratentorial ependymomas. Clin. Cancer Res..

[115] Queisser A., Hagedorn S., Wang H., Schaefer T., Konantz M., Alavi S., Deng M., Vogel W., von Mässenhausen A., Kristiansen G. (2017). Ecotropic viral integration site 1, a novel oncogene in prostate cancer. Oncogene.

[116] Wang H., Schaefer T., Konantz M., Braun M., Varga Z., Paczulla A.M., Reich S., Jacob F., Perner S., Moch H. (2017). Prominent Oncogenic Roles of EVI1 in Breast Carcinoma. Cancer Res..

[117] Kurokawa M., Mitani K., Irie K., Matsuyama T., Takahashi T., Chiba S., Yazaki Y., Matsumoto K., Hirai H. (1998). The oncoprotein Evi-1 represses TGF-beta signalling by inhibiting Smad3. Nature.

[118] Yoshimi A., Kurokawa M. (2011). Evi1 forms a bridge between the epigenetic machinery and signaling pathways. Oncotarget.

[119] Zhou L.Y., Chen F.Y., Shen L.J., Wan H.X., Zhong J.H. (2014). Arsenic trioxide induces apoptosis in the THP1 cell line by downregulating EVI-1. Exp. Ther. Med..

[120] Kurokawa M., Mitani K., Yamagata T., Takahashi T., Izutsu K., Ogawa S., Moriguchi T., Nishida E., Yazaki Y., Hirai H. (2000). The evi-1 oncoprotein inhibits c-Jun N-terminal kinase and prevents stress-induced cell death. EMBO J..

[121] Yatsula B., Lin S., Read A.J., Poholek A., Yates K., Yue D., Hui P., Perkins A.S. (2005). Identification of binding sites of EVI1 in mammalian cells. J. Biol. Chem..

[122] Yuasa H., Oike Y., Iwama A., Nishikata I., Sugiyama D., Perkins A., Mucenski M.L., Suda T., Morishita K. (2005). Oncogenic transcription factor Evi1 regulates hematopoietic stem cell proliferation through GATA-2 expression. EMBO J..

[123] Nayak K.B., Sajitha I.S., Kumar T.R.S., Chakraborty S. (2018). Ecotropic viral integration site 1 promotes metastasis independent of epithelial mesenchymal transition in colon cancer cells. Cell Death Dis..

[124] Lu Y., Liang Y., Zheng X., Deng X., Huang W., Zhang G. (2019). EVI1 promotes epithelial-to-mesenchymal transition, cancer stem cell features and chemo-/radioresistance in nasopharyngeal carcinoma. J. Exp. Clin. Cancer Res..

[125] Bard-Chapeau E.A., Jeyakani J., Kok C.H., Muller J., Chua B.Q., Gunaratne J., Batagov A., Jenjaroenpun P., Kuznetsov V.A., Wei C.L. (2012). Ecotopic viral integration site 1 (EVI1) regulates multiple cellular processes important for cancer and is a synergistic partner for FOS protein in invasive tumors. Proc. Natl. Acad. Sci. USA.

[126] Ripperger T., Hofmann W., Koch J.C., Shirneshan K., Haase D., Wulf G., Issing P.R., Karnebogen M., Schmidt G., Auber B. (2018). MDS1 and EVI1 complex locus (MECOM): A novel candidate gene for hereditary hematological malignancies. Haematologica.

[127] Choi E.J., Kim M.S., Song S.Y., Yoo N.J., Lee S.H. (2017). Intratumoral Heterogeneity of Frameshift Mutations in MECOM Gene is Frequent in Colorectal Cancers with High Microsatellite Instability. Pathol. Oncol. Res..

[128] Yang X.H., Huang S. (1999). PFM1 (PRDM4), a new member of the PR-domain family, maps to a tumor suppressor locus on human chromosome 12q23-q24.1. Genomics.

[129] Yan Z., Xiong Y., Xu W., Li M., Cheng Y., Chen F., Ding S., Xu H., Zheng G. (2012). Identification of recurrence-related genes by integrating microRNA and gene expression profiling of gastric cancer. Int. J. Oncol..

[130] Liu H., Dai X., Cao X., Yan H., Ji X., Zhang H., Shen S., Si Y., Zhang H., Chen J. (2018). PRDM4 mediates YAP-induced cell invasion by activating leukocyte-specific integrin β2 expression. EMBO Rep..

[131] Deng Q., Huang S. (2004). PRDM5 is silenced in human cancers and has growth suppressive activities. Oncogene.

[132] Watanabe Y., Toyota M., Kondo Y., Suzuki H., Imai T., Ohe-Toyota M., Maruyama R., Nojima M., Sasaki Y., Sekido Y. (2007). PRDM5 identified as a target of epigenetic silencing in colorectal and gastric cancer. Clin. Cancer Res..

[133] Tahara S., Tahara T., Horiguchi N., Kato T., Shinkai Y., Yamashita H., Yamada H., Kawamura T., Terada T., Okubo M. (2019). DNA methylation accumulation in gastric mucosa adjacent to cancer after Helicobacter pylori eradication. Int. J. Cancer.

[134] Galli G.G., Multhaupt H.A., Carrara M., de Lichtenberg K.H., Christensen I.B., Santoni-Rugiu E., Calogero R.A., Lund A.H. (2014). Prdm5 suppresses Apc(Min)-driven intestinal adenomas and regulates monoacylglycerol lipase expression. Oncogene.

[135] Bond C.E., Bettington M.L., Pearson S.A., McKeone D.M., Leggett B.A., Whitehall V.L. (2015). Methylation and expression of the tumour suppressor, PRDM5, in colorectal cancer and polyp subgroups. BMC Cancer.

[136] Cheng H.Y., Chen X.W., Cheng L., Liu Y.D., Lou G. (2010). DNA methylation and carcinogenesis of PRDM5 in cervical cancer. J. Cancer Res. Clin. Oncol..

[137] Shu X.S., Geng H., Li L., Ying J., Ma C., Wang Y., Poon F.F., Wang X., Ying Y., Yeo W. (2011). The epigenetic modifier PRDM5 functions as a tumor suppressor through modulating WNT/β-catenin signaling and is frequently silenced in multiple tumors. PLoS ONE.

[138] Tan S.X., Hu R.C., Tan Y.L., Liu J.J., Liu W.E. (2014). Promoter methylation-mediated downregulation of PRDM5 contributes to the development of lung squamous cell carcinoma. Tumour Biol..

[139] Tan S.X., Hu R.C., Liu J.J., Tan Y.L., Liu W.E. (2014). Methylation of PRDM2, PRDM5 and PRDM16 genes in lung cancer cells. Int. J. Clin. Exp. Pathol..

[140] Tan S.X., Hu R.C., Xia Q., Tan Y.L., Liu J.J., Gan G.X., Wang L.L. (2018). The methylation profiles of PRDM promoters in non-small cell lung cancer. Onco. Targets Ther..

[141] Marzec-Kotarska B., Cybulski M., Kotarski J.C., Ronowicz A., Tarkowski R., Polak G., Antosz H., Piotrowski A., Kotarski J. (2016). Molecular bases of aberrant miR-182 expression in ovarian cancer. Genes Chromosomes Cancer.

[142] Seehawer M., Heinzmann F., D’Artista L., Harbig J., Roux P.F., Hoenicke L., Dang H., Klotz S., Robinson L., Doré G. (2018). Necroptosis microenvironment directs lineage commitment in liver cancer. Nature.

[143] Wang L., Ding Q.Q., Gao S.S., Yang H.J., Wang M., Shi Y., Cheng B.F., Bi J.J., Feng Z.W. (2016). PRDM5 promotes the proliferation and invasion of murine melanoma cells through up-regulating JNK expression. Cancer Med..

[144] Armengol G., Eissa S., Lozano J.J., Shoman S., Sumoy L., Caballín M.R., Knuutila S. (2007). Genomic imbalances in Schistosoma-associated and non-Schistosoma-associated bladder carcinoma. An array comparative genomic hybridization analysis. Cancer Genet. Cytogenet..

[145] Lindström S., Thompson D.J., Paterson A.D., Li J., Gierach G.L., Scott C., Stone J., Douglas J.A., dos-Santos-Silva I., Fernandez-Navarro P. (2014). Genome-wide association study identifies multiple loci associated with both mammographic density and breast cancer risk. Nat. Commun..

[146] Northcott P.A., Buchhalter I., Morrissy A.S., Hovestadt V., Weischenfeldt J., Ehrenberger T., Gröbner S., Segura-Wang M., Zichner T., Rudneva V.A. (2017). The whole-genome landscape of medulloblastoma subtypes. Nature.

[147] Basso T.R., Villacis R.A., Canto L.M., Alves V.M., Lapa R.M., Nóbrega A.F., Achatz M.I., Rogatto S.R. (2015). Genomic profile of a Li-Fraumeni-like syndrome patient with a 45,X/46,XX karyotype, presenting neither mutations in TP53 nor clinical stigmata of Turner syndrome. Cancer Genet..

[148] Lan X., Gao H., Wang F., Feng J., Bai J., Zhao P., Cao L., Gui S., Gong L., Zhang Y. (2016). Whole-exome sequencing identifies variants in invasive pituitary adenomas. Oncol. Lett..

[149] Wu X., Miao J., Jiang J., Liu F. (2017). Analysis of methylation profiling data of hyperplasia and primary and metastatic endometrial cancers. Eur. J. Obstet. Gynecol. Reprod. Biol..

[150] Chen Z., Gao W., Pu L., Zhang L., Han G., Zuo X., Zhang Y., Li X., Shen H., Wu J. (2018). PRDM8 exhibits antitumor activities toward hepatocellular carcinoma by targeting NAP1L1. Hepatology.

[151] Paigen K., Petkov P.M. (2018). PRDM9 and Its Role in Genetic Recombination. Trends Genet..

[152] Alves I., Houle A.A., Hussin J.G., Awadalla P. (2017). The impact of recombination on human mutation load and disease. Philos. Trans. R. Soc. Lond. B. Biol. Sci..

[153] Altemose N., Noor N., Bitoun E., Tumian A., Imbeault M., Chapman J.R., Aricescu A.R., Myers S.R. (2017). A map of human PRDM9 binding provides evidence for novel behaviors of PRDM9 and other zinc-finger proteins in meiosis. eLife.

[154] Feichtinger J., Aldeailej I., Anderson R., Almutairi M., Almatrafi A., Alsiwiehri N., Griffiths K., Stuart N., Wakeman J.A., Larcombe L. (2012). Meta-analysis of clinical data using human meiotic genes identifies a novel cohort of highly restricted cancer-specific marker genes. Oncotarget.

[155] Hussin J., Sinnett D., Casals F., Idaghdour Y., Bruat V., Saillour V., Healy J., Grenier J.C., de Malliard T., Busche S. (2013). Rare allelic forms of PRDM9 associated with childhood leukemogenesis. Genome Res..

[156] Woodward E.L., Olsson M.L., Johansson B., Paulsson K. (2014). Allelic variants of PRDM9 associated with high hyperdiploid childhood acute lymphoblastic leukaemia. Br. J. Haematol..

[157] Spinella J.F., Healy J., Saillour V., Richer C., Cassart P., Ouimet M., Sinnett D. (2015). Whole-exome sequencing of a rare case of familial childhood acute lymphoblastic leukemia reveals putative predisposing mutations in Fanconi anemia genes. BMC Cancer.

[158] Zou A.E., Zheng H., Saad M.A., Rahimy M., Ku J., Kuo S.Z., Honda T.K., Wang-Rodriguez J., Xuan Y., Korrapati A. (2016). The non-coding landscape of head and neck squamous cell carcinoma. Oncotarget.

[159] Ding B., Yan L., Zhang Y., Wang Z., Zhang Y., Xia D., Ye Z., Xu H. (2019). Analysis of the role of mutations in the KMT2D histone lysine methyltransferase in bladder cancer. FEBS Open Bio.

[160] Houle A.A., Gibling H., Lamaze F.C., Edgington H.A., Soave D., Fave M.J., Agbessi M., Bruat V., Stein L.D., Awadalla P. (2018). Aberrant PRDM9 expression impacts the pan-cancer genomic landscape. Genome Res..

[161] Hofvander J., Tayebwa J., Nilsson J., Magnusson L., Brosjö O., Larsson O., Vult von Steyern F., Mandahl N., Fletcher C.D., Mertens F. (2015). Recurrent PRDM10 gene fusions in undifferentiated pleomorphic sarcoma. Clin. Cancer Res..

[162] Puls F., Pillay N., Fagman H., Palin-Masreliez A., Amary F., Hansson M., Kindblom L.G., McCulloch T.A., Meligonis G., Muc R. (2019). PRDM10-rearrangedSoft Tissue Tumor: A Clinicopathologic Study of 9 Cases. Am. J. Surg. Pathol..

[163] Lou W., Liu J., Ding B., Xu L., Fan W. (2018). Identification of chemoresistance-associated miRNAs in breast cancer. Cancer Manag. Res..

[164] Mansouri V., Rezaei Tavirani S., Zadeh-Esmaeel M.M., Rostami-Nejad M., Rezaei-Tavirani M. (2018). Comparative study of gastric cancer and chronic gastritis via network analysis. Gastroenterol. Hepatol. Bed Bench.

[165] Rostami-Nejad M., Mansouri V., Mahmoud Robati R., Mohaghegh Shalmani H., Mahmoudi Lamouki R., Rezaei Tavirani M. (2018). Network analysis of grade II into grade III transition in rectum cancer patients. Gastroenterol. Hepatol. Bed Bench.

[166] Wu K., Yin X., Jin Y., Liu F., Gao J. (2019). Identification of aberrantly methylated differentially expressed genes in prostate carcinoma using integrated bioinformatics. Cancer Cell Int..

[167] Zamanian Azodi M., Rezaei Tavirani M., Rezaei Tavirani M., Vafaee R., Rostami-Nejad M. (2018). Nasopharyngeal Carcinoma Protein Interaction Mapping Analysis via Proteomic Approaches. Asian Pac. J. Cancer Prev..

[168] Zhang L., Huang Y., Ling J., Zhuo W., Yu Z., Shao M., Luo Y., Zhu Y. (2018). Screening and function analysis of hub genes and pathways in hepatocellular carcinoma via bioinformatics approaches. Cancer Biomark..

[169] Chen N., Hu T., Gui Y., Gao J., Li Z., Huang S. (2019). Transcriptional regulation of Bcl-2 gene by the PR/SET domain family member PRDM10. PeerJ.

[170] Chen C., Bartenhagen C., Gombert M., Okpanyi V., Binder V., Röttgers S., Bradtke J., Teigler-Schlegel A., Harbott J., Ginzel S. (2013). Next-generation-sequencing-based risk stratification and identification of new genes involved in structural and sequence variations in near haploid lymphoblastic leukemia. Genes Chromosomes Cancer.

[171] Fog C.K., Asmar F., Côme C., Jensen K.T., Johansen J.V., Kheir T.B., Jacobsen L., Friis C., Louw A., Rosgaard L. (2015). Loss of PRDM11 promotes MYC-driven lymphomagenesis. Blood.

[172] Wei Y., Chang Z., Wu C., Zhu Y., Li K., Xu Y. (2017). Identification of potential cancer-related pseudogenes in lung adenocarcinoma based on ceRNA hypothesis. Oncotarget.

[173] Kolomietz E., Marrano P., Yee K., Thai B., Braude I., Kolomietz A., Chun K., Minkin S., Kamel-Reid S., Minden M. (2003). Quantitative PCR identifies a minimal deleted region of 120 kb extending from the Philadelphia chromosome ABL translocation breakpoint in chronic myeloid leukemia with poor outcome. Leukemia.

[174] Reid A.G., Nacheva E.P. (2004). A potential role for PRDM12 in the pathogenesis of chronic myeloid leukaemia with derivative chromosome 9 deletion. Leukemia.

[175] Huet S., Dulucq S., Chauveau A., Ménard A., Chomel J.C., Maisonneuve H., Legros L., Perrin M.C., Ferrant E., Moreilhon C. (2015). Molecular characterization and follow-up of five CML patients with new BCR-ABL1 fusion transcripts. Genes Chromosomes Cancer.

[176] Zhang Y., Yan L., Yao W., Chen K., Xu H., Ye Z. (2019). Integrated Analysis of Genetic Abnormalities of the Histone Lysine Methyltransferases in Prostate Cancer. Med. Sci. Monit..

[177] Chen Y.C., Auer-Grumbach M., Matsukawa S., Zitzelsberger M., Themistocleous A.C., Strom T.M., Samara C., Moore A.W., Cho L.T., Young G.T. (2015). Transcriptional regulator PRDM12 is essential for human pain perception. Nat. Genet..

[178] Behrends U., Schneider I., Rössler S., Frauenknecht H., Golbeck A., Lechner B., Eigenstetter G., Zobywalski C., Müller-Weihrich S., Graubner U. (2003). Novel tumor antigens identified by autologous antibody screening of childhood medulloblastoma cDNA libraries. Int. J. Cancer.

[179] Rubicz R., Zhao S., Geybels M., Wright J.L., Kolb S., Klotzle B., Bibikova M., Troyer D., Lance R., Ostrander E.A. (2019). DNA methylation profiles in African American prostate cancer patients in relation to disease progression. Genomics.

[180] Zhang L., Cao H., He T., Yang J., Tao H., Wang Y., Hu Q. (2018). Overexpression of PRDM13 inhibits glioma cells via Rho and GTP enzyme activation protein. Int. J. Mol. Med..

[181] Nishikawa N., Toyota M., Suzuki H., Honma T., Fujikane T., Ohmura T., Nishidate T., Ohe-Toyota M., Maruyama R., Sonoda T. (2007). Gene amplification and overexpression of PRDM14 in breast cancers. Cancer Res..

[182] Moelans C.B., de Weger R.A., Monsuur H.N., Vijzelaar R., van Diest P.J. (2010). Molecular profiling of invasive breast cancer by multiplex ligation-dependent probe amplification-based copy number analysis of tumor suppressor and oncogenes. Mod. Pathol..

[183] Moelans C.B., de Wegers R.A., Monsuurs H.N., Maess A.H., van Diest P.J. (2011). Molecular differences between ductal carcinoma in situ and adjacent invasive breast carcinoma: A multiplex ligation-dependent probe amplification study. Cell Oncol..

[184] Moelans C.B., van der Groep P., Hoefnagel L.D.C., van de Vijver M.J., Wesseling P., Wesseling J., van der Wall E., van Diest P.J. (2014). Genomic evolution from primary breast carcinoma to distant metastasis: Few copy number changes of breast cancer related genes. Cancer Lett..

[185] Seki Y. (2018). PRDM14 Is a Unique Epigenetic Regulator Stabilizing Transcriptional Networks for Pluripotency. Front. Cell Dev. Biol..

[186] Taniguchi H., Hoshino D., Moriya C., Zembutsu H., Nishiyama N., Yamamoto H., Kataoka K., Imai K. (2017). Silencing PRDM14 expression by an innovative RNAi therapy inhibits stemness, tumorigenicity, and metastasis of breast cancer. Oncotarget.

[187] Ou M., Li S., Tang L. (2018). PRDM14: A Potential Target for Cancer Therapy. Curr. Cancer Drug Targets.

[188] Nandy S.B., Orozco A., Lopez-Valdez R., Roberts R., Subramani R., Arumugam A., Dwivedi A.K., Stewart V., Prabhakar G., Jones S. (2017). Glucose insult elicits hyperactivation of cancer stem cells through miR-424-cdc42-prdm14 signalling axis. Br. J. Cancer.

[189] Moriya C., Taniguchi H., Nagatoishi S., Igarashi H., Tsumoto K., Imai K. (2018). PRDM14 directly interacts with heat shock proteins HSP90α and glucose-regulated protein 78. Cancer Sci..

[190] Dettman E.J., Justice M.J. (2008). The zinc finger SET domain gene Prdm14 is overexpressed in lymphoblastic lymphomas with retroviral insertions at Evi32. PLoS ONE.

[191] Dettman E.J., Simko S.J., Ayanga B., Carofino B.L., Margolin J.F., Morse H.C., Justice M.J. (2011). Prdm14 initiates lymphoblastic leukemia after expanding a population of cells resembling common lymphoid progenitors. Oncogene.

[192] Simko S.J., Voicu H., Carofino B.L., Justice M.J. (2012). Mouse Lymphoblastic Leukemias Induced by Aberrant Prdm14 Expression Demonstrate Widespread Copy Number Alterations Also Found in Human ALL. Cancers.

[193] Carofino B.L., Ayanga B., Justice M.J. (2013). A mouse model for inducible overexpression of Prdm14 results in rapid-onset and highly penetrant T-cell acute lymphoblastic leukemia (T-ALL). Dis. Model. Mech..

[194] Carofino B.L., Ayanga B., Tracey L.J., Brooke-Bisschop T., Justice M.J. (2016). PRDM14 promotes RAG-dependent Notch1 driver mutations in mouse T-ALL. Biol. Open.

[195] Tracey L.J., Brooke-Bisschop T., Jansen P.W.T.C., Campos E.I., Vermeulen M., Justice M.J. (2019). The pluripotency regulator PRDM14 requires hematopoietic regulator CBFA2T3 to initiate leukemia in mice. Mol. Cancer Res..

[196] Zhang T., Meng L., Dong W., Shen H., Zhang S., Liu Q., Du J. (2013). High expression of PRDM14 correlates with cell differentiation and is a novel prognostic marker in resected non-small cell lung cancer. Med. Oncol..

[197] Bi H.X., Shi H.B., Zhang T., Cui G. (2015). PRDM14 promotes the migration of human non-small cell lung cancer through extracellular matrix degradation in vitro. Chin. Med. J..

[198] Lu Y., Wan Z., Zhang X., Zhong X., Rui L., Li Z. (2016). PRDM14 inhibits 293T cell proliferation by influencing the G1/S phase transition. Gene.

[199] Baykara O., Bakir B., Buyru N., Kaynak K., Dalay N. (2015). Amplification of chromosome 8 genes in lung cancer. J. Cancer.

[200] Moriya C., Taniguchi H., Miyata K., Nishiyama N., Kataoka K., Imai K. (2017). Inhibition of PRDM14 expression in pancreatic cancer suppresses cancer stem-like properties and liver metastasis in mice. Carcinogenesis.

[201] Moriya C., Imai K., Taniguchi H. (2018). PRDM14 is overexpressed in chronic pancreatitis prior to pancreatic cancer. FEBS Open Bio.

[202] Terashima K., Yu A., Chow W.Y., Hsu W.C., Chen P., Wong S., Hung Y.S., Suzuki T., Nishikawa R., Matsutani M. (2014). Genome-wide analysis of DNA copy number alterations and loss of heterozygosity in intracranial germ cell tumors. Pediatr. Blood Cancer.

[203] Baltaci E., Karaman E., Dalay N., Buyru N. (2018). Analysis of gene copy number changes in head and neck cancer. Clin. Otolaryngol..

[204] Ruark E., Seal S., McDonald H., Zhang F., Elliot A., Lau K., Perdeaux E., Rapley E., Eeles R., Peto J. (2013). Identification of nine new susceptibility loci for testicular cancer, including variants near DAZL and PRDM14. Nat. Genet..

[205] Gell J.J., Zhao J., Chen D., Hunt T.J., Clark A.T. (2018). PRDM14 is expressed in germ cell tumors with constitutive overexpression altering human germline differentiation and proliferation. Stem Cell Res..

[206] Steenbergen R.D., Ongenaert M., Snellenberg S., Trooskens G., van der Meide W.F., Pandey D., Bloushtain-Qimron N., Polyak K., Meijer C.J., Snijders P.J. (2013). Methylation-specific digital karyotyping of HPV16E6E7-expressing human keratinocytes identifies novel methylation events in cervical carcinogenesis. J. Pathol..

[207] Snellenberg S., Cillessen S.A., Van Criekinge W., Bosch L., Meijer C.J., Snijders P.J., Steenbergen R.-D. (2014). Methylation-mediated repression of PRDM14 contributes to apoptosis evasion in HPV-positive cancers. Carcinogenesis.

[208] Kitchen M.O., Bryan R.T., Emes R.D., Glossop J.R., Luscombe C., Cheng K.K., Zeegers M.P., James N.D., Devall A.J., Mein C.A. (2016). Quantitative genome-wide methylation analysis of high-grade non-muscle invasive bladder cancer. Epigenetics.

[209] Ashktorab H., Shakoori A., Zarnogi S., Sun X., Varma S., Lee E., Shokrani B., Laiyemo A.O., Washington K., Brim H. (2016). Reduced Representation Bisulfite Sequencing Determination of Distinctive DNA Hypermethylated Genes in the Progression to Colon Cancer in African Americans. Gastroenterol. Res. Pract..

[210] Hubers A.J., Brinkman P., Boksem R.J., Rhodius R.J., Witte B.I., Zwinderman A.H., Heideman D.A., Duin S., Koning R., Steenbergen R.D. (2014). Combined sputum hypermethylation and eNose analysis for lung cancer diagnosis. J. Clin. Pathol..

[211] Hubers A.J., Heideman D.A., Burgers S.A., Herder G.J., Sterk P.J., Rhodius R.J., Smit H.J., Krouwels F., Welling A., Witte B.I. (2015). DNA hypermethylation analysis in sputum for the diagnosis of lung cancer: Training validation set approach. Br. J. Cancer.

[212] Su Y., Fang H., Jiang F. (2016). Integrating DNA methylation and microRNA biomarkers in sputum for lung cancer detection. Clin. Epigenetics.

[213] Bashyam M.D., Bair R., Kim Y.H., Wang P., Hernandez-Boussard T., Karikari C.A., Tibshirani R., Maitra A., Pollack J.R. (2005). Array-based comparative genomic hybridization identifies localized DNA amplifications and homozygous deletions in pancreatic cancer. Neoplasia.

[214] Giallourakis C.C., Benita Y., Molinie B., Cao Z., Despo O., Pratt H.E., Zukerberg L.R., Daly M.J., Rioux J.D., Xavier R.J. (2013). Genome-wide analysis of immune system genes by expressed sequence Tag profiling. J. Immunol..

[215] Park H.Y., Lee S.B., Yoo H.Y., Kim S.J., Kim W.S., Kim J.I., Ko Y.H. (2016). Whole-exome and transcriptome sequencing of refractory diffuse large B-cell lymphoma. Oncotarget.

[216] Mzoughi S., Zhang J., Hequet D., Teo S.X., Fang H., Xing Q.R., Bezzi M., Seah M.K.Y., Ong S.L.M., Shin E.M. (2017). PRDM15 safeguards naive pluripotency by transcriptionally regulating WNT and MAPK-ERK signaling. Nat. Genet..

[217] Mochizuki N., Shimizu S., Nagasawa T., Tanaka H., Taniwaki M., Yokota J., Morishita K. (2000). A novel gene, MEL1, mapped to 1p36.3 is highly homologous to the MDS1/EVI1 gene and is transcriptionally activated in t(1;3)(p36;q21)-positive leukemia cells. Blood.

[218] Nishikata I., Sasaki H., Iga M., Tateno Y., Imayoshi S., Asou N., Nakamura T., Morishita K. (2003). A novel EVI1 gene family, MEL1, lacking a PR domain (MEL1S) is expressed mainly in t(1;3)(p36;q21)-positive AML and blocks G-CSF-induced myeloid differentiation. Blood.

[219] Yoshida M., Nosaka K., Yasunaga J., Nishikata I., Morishita K., Matsuoka M. (2004). Aberrant expression of the MEL1S gene identified in association with hypomethylation in adult T-cell leukemia cells. Blood.

[220] Shing D.C., Trubia M., Marchesi F., Radaelli E., Belloni E., Tapinassi C., Scanziani E., Mecucci C., Crescenzi B., Lahortiga I. (2007). Overexpression of sPRDM16 coupled with loss of p53 induces myeloid leukemias in mice. J. Clin. Investig..

[221] Sakai I., Tamura T., Narumi H., Uchida N., Yakushijin Y., Hato T., Fujita S., Yasukawa M. (2005). Novel RUNX1-PRDM16 fusion transcripts in a patient with acute myeloid leukemia showing t(1;21)(p36;q22). Genes Chromosomes Cancer.

[222] Stevens-Kroef M.J., Schoenmakers E.F., van Kraaij M., Huys E., Vermeulen S., van der Reijden B., van Kessel A.G. (2006). Identification of truncated RUNX1 and RUNX1-PRDM16 fusion transcripts in a case of t(1;21)(p36;q22)-positive therapy-related AML. Leukemia.

[223] Hazourli S., Chagnon P., Sauvageau M., Fetni R., Busque L., Hébert J. (2006). Overexpression of PRDM16 in the presence and absence of the RUNX1/PRDM16 fusion gene in myeloid leukemias. Genes Chromosomes Cancer.

[224] Roche-Lestienne C., Deluche L., Corm S., Tigaud I., Joha S., Philippe N., Geffroy S., Laï J.L., Nicolini F.E., Preudhomme C. (2008). RUNX1 DNA-binding mutations and RUNX1-PRDM16 cryptic fusions in BCR-ABL+ leukemias are frequently associated with secondary trisomy 21 and may contribute to clonal evolution and imatinib resistance. Blood.

[225] Deluche L., Joha S., Corm S., Daudignon A., Geffroy S., Quief S., Villenet C., Kerckaert J.P., Laï J.L., Preudhomme C. (2008). Cryptic and partial deletions of PRDM16 and RUNX1 without t(1;21)(p36;q22) and/or RUNX1-PRDM16 fusion in a case of progressive chronic myeloid leukemia: A complex chromosomal rearrangement of underestimated frequency in disease progression?. Genes Chromosomes Cancer.

[226] Xinh P.T., Tri N.K., Nagao H., Nakazato H., Taketazu F., Fujisawa S., Yagasaki F., Chen Y.Z., Hayashi Y., Toyoda A. (2003). Breakpoints at 1p36.3 in three MDS/AML(M4) patients with t(1;3)(p36;q21) occur in the first intron and in the 5’ region of MEL1. Genes Chromosomes Cancer.

[227] Wong K.F., Wong M.L., Tu S.P. (2006). Dup(1)(p31.2p36.2) in acute myelomonocytic leukemia. Cancer Genet. Cytogenet..

[228] Storlazzi C.T., Albano F., Guastadisegni M.C., Impera L., Mühlematter D., Meyer-Monard S., Wuillemin W., Rocchi M., Jotterand M. (2008). Upregulation of MEL1 and FLJ42875 genes by position effect resulting from a t(1;2)(p36;p21) occurring during evolution of chronic myelomonocytic leukemia. Blood Cells Mol. Dis..

[229] Quentin S., Cuccuini W., Ceccaldi R., Nibourel O., Pondarre C., Pagès M.P., Vasquez N., Dubois d’Enghien C., Larghero J., Peffault de Latour R. (2011). Myelodysplasia and leukemia of Fanconi anemia are associated with a specific pattern of genomic abnormalities that includes cryptic RUNX1/AML1 lesions. Blood.

[230] Duhoux F.P., Ameye G., Montano-Almendras C.P., Bahloula K., Mozziconacci M.J., Laibe S., Wlodarska I., Michaux L., Talmant P., Richebourg S. (2012). PRDM16 (1p36) translocations define a distinct entity of myeloid malignancies with poor prognosis but may also occur in lymphoid malignancies. Br. J. Haematol..

[231] Masetti R., Togni M., Astolfi A., Pigazzi M., Indio V., Rivalta B., Manara E., Rutella S., Basso G., Pession A. (2014). Whole transcriptome sequencing of a paediatric case of de novo acute myeloid leukaemia with del(5q) reveals RUNX1-USP42 and PRDM16-SKI fusion transcripts. Br. J. Haematol..

[232] Jo A., Mitani S., Shiba N., Hayashi Y., Hara Y., Takahashi H., Tsukimoto I., Tawa A., Horibe K., Tomizawa D. (2015). High expression of EVI1 and MEL1 is a compelling poor prognostic marker of pediatric AML. Leukemia.

[233] Shiba N., Ohki K., Kobayashi T., Hara Y., Yamato G., Tanoshima R., Ichikawa H., Tomizawa D., Park M.J., Shimada A. (2016). High PRDM16 expression identifies a prognostic subgroup of pediatric acute myeloid leukaemia correlated to FLT3-ITD, KMT2A-PTD, and NUP98-NSD1: The results of the Japanese Paediatric Leukaemia/Lymphoma Study Group AML-05 trial. Br. J. Haematol..

[234] Yamato G., Yamaguchi H., Handa H., Shiba N., Kawamura M., Wakita S., Inokuchi K., Hara Y., Ohki K., Okubo J. (2017). Clinical features and prognostic impact of PRDM16 expression in adult acute myeloid leukemia. Genes Chromosomes Cancer.

[235] Miyamura T., Moritake H., Nakayama H., Tanaka S., Tomizawa D., Shiba N., Saito A.M., Tawa A., Shimada A., Iwamoto S. (2019). Clinical and biological features of paediatric acute myeloid leukaemia (AML) with primary induction failure in the Japanese Paediatric Leukaemia/Lymphoma Study Group AML-05 study. Br. J. Haematol..

[236] Barjesteh van Waalwijk van Doorn-Khosrovani S., Erpelinck C., Löwenberg B., Delwel R. (2003). Low expression of MDS1-EVI1-like-1 (MEL1) and EVI1-like-1 (EL1) genes in favorable-risk acute myeloid leukemia. Exp. Hematol..

[237] Yu H., Neale G., Zhang H., Lee H.M., Ma Z., Zhou S., Forget B.G., Sorrentino B.P. (2014). Downregulation of Prdm16 mRNA is a specific antileukemic mechanism during HOXB4-mediated HSC expansion in vivo. Blood.

[238] Dong S., Chen J. (2015). SUMOylation of sPRDM16 promotes the progression of acute myeloid leukemia. BMC Cancer.

[239] Du Y., Jenkins N.A., Copeland N.G. (2005). Insertional mutagenesis identifies genes that promote the immortalization of primary bone marrow progenitor cells. Blood.

[240] Ott M.G., Schmidt M., Schwarzwaelder K., Stein S., Siler U., Koehl U., Glimm H., Kühlcke K., Schilz A., Kunkel H. (2006). Correction of X-linked chronic granulomatous disease by gene therapy, augmented by insertional activation of MDS1-EVI1, PRDM16 or SETBP1. Nat. Med..

[241] Adair J.E., Beard B.C., Trobridge G.D., Neff T., Rockhill J.K., Silbergeld D.L., Mrugala M.M., Kiem H.P. (2012). Extended survival of glioblastoma patients after chemoprotective HSC gene therapy. Sci. Transl. Med..

[242] Matsuo H., Goyama S., Kamikubo Y., Adachi S. (2015). The subtype-specific features of EVI1 and PRDM16 in acute myeloid leukemia. Haematologica.

[243] Eveillard M., Delaunay J., Richebourg S., Lodé L., Garand R., Wuillème S., Duhoux F., Antoine-Poirel H., Godon C., Béné M.C. (2015). The closely related rare and severe acute myeloid leukemias carrying EVI1 or PRDM16 rearrangements share singular biological features. Haematologica.

[244] Corrigan D.J., Luchsinger L.L., Justino de Almeida M., Williams L.J., Strikoudis A., Snoeck H.W. (2018). PRDM16 isoforms differentially regulate normal and leukemic hematopoiesis and inflammatory gene signature. J. Clin. Investig..

[245] Ivanochko D., Halabelian L., Henderson E., Savitsky P., Jain H., Marcon E., Duan S., Hutchinson A., Seitova A., Barsyte-Lovejoy D. (2019). Direct interaction between the PRDM3 and PRDM16 tumor suppressors and the NuRD chromatin remodeling complex. Nucleic Acids Res..

[246] Man T.K., Lu X.Y., Jaeweon K., Perlaky L., Harris C.P., Shah S., Ladanyi M., Gorlick R., Lau C.C., Rao P.H. (2004). Genome-wide array comparative genomic hybridization analysis reveals distinct amplifications in osteosarcoma. BMC Cancer.

[247] Beck A.H., Lee C.H., Witten D.M., Gleason B.C., Edris B., Espinosa I., Zhu S., Li R., Montgomery K.D., Marinelli R.J. (2010). Discovery of molecular subtypes in leiomyosarcoma through integrative molecular profiling. Oncogene.

[248] Cuppens T., Moisse M., Depreeuw J., Annibali D., Colas E., Gil-Moreno A., Huvila J., Carpén O., Zikán M., Matias-Guiu X. (2018). Integrated genome analysis of uterine leiomyosarcoma to identify novel driver genes and targetable pathways. Int. J. Cancer.

[249] Burghel G.J., Lin W.Y., Whitehouse H., Brock I., Hammond D., Bury J., Stephenson Y., George R., Cox A. (2013). Identification of candidate driver genes in common focal chromosomal aberrations of microsatellite stable colorectal cancer. PLoS ONE.

[250] Mehrian-Shai R., Yalon M., Moshe I., Barshack I., Nass D., Jacob J., Dor C., Reichardt J.K., Constantini S., Toren A. (2016). Identification of genomic aberrations in hemangioblastoma by droplet digital PCR and SNP microarray highlights novel candidate genes and pathways for pathogenesis. BMC Genom..

[251] Takahata M., Inoue Y., Tsuda H., Imoto I., Koinuma D., Hayashi M., Ichikura T., Yamori T., Nagasaki K., Yoshida M. (2009). SKI and MEL1 cooperate to inhibit transforming growth factor-beta signal in gastric cancer cells. J. Biol. Chem..

[252] Bibi F., Ali I., Naseer M.I., Ali Mohamoud H.S., Yasir M., Alvi S.A., Jiman-Fatani A.A., Sawan A., Azhar E.I. (2018). Detection of genetic alterations in gastric cancer patients from Saudi Arabia using comparative genomic hybridization (CGH). PLoS ONE.

[253] Yang L., Zhang W., Wang Y., Zou T., Zhang B., Xu Y., Pang T., Hu Q., Chen M., Wang L. (2018). Hypoxia-induced miR-214 expression promotes tumour cell proliferation and migration by enhancing the Warburg effect in gastric carcinoma cells. Cancer Lett..

[254] Zhang D.L., Qu L.W., Ma L., Zhou Y.C., Wang G.Z., Zhao X.C., Zhang C., Zhang Y.F., Wang M., Zhang M.Y. (2018). Genome-wide identification of transcription factors that are critical to non-small cell lung cancer. Cancer Lett..

[255] Lv W., Yu X., Li W., Feng N., Feng T., Wang Y., Lin H., Qian B. (2018). Low expression of LINC00982 and PRDM16 is associated with altered gene expression, damaged pathways and poor survival in lung adenocarcinoma. Oncol. Rep..

[256] Fei L.R., Huang W.J., Wang Y., Lei L., Li Z.H., Zheng Y.W., Wang Z., Yang M.Q., Liu C.C., Xu H.T. (2019). PRDM16 functions as a suppressor of lung adenocarcinoma metastasis. J. Exp. Clin. Cancer Res..

[257] Lei Q., Liu X., Fu H., Sun Y., Wang L., Xu G., Wang W., Yu Z., Liu C., Li P. (2016). miR-101 reverses hypomethylation of the PRDM16 promoter to disrupt mitochondrial function in astrocytoma cells. Oncotarget.

[258] Li P., Wu M., De Vleeschouwer S. (2017). Epigenetic Mechanisms of Glioblastoma. Glioblastoma.

[259] Peng X., Xue H., Lü L., Shi P., Wang J., Wang J. (2017). Accumulated promoter methylation as a potential biomarker for esophageal cancer. Oncotarget.

[260] Deng J., Kong W., Mou X., Wang S., Zeng W. (2018). Identifying novel candidate biomarkers of RCC based on WGCNA analysis. Pers. Med..

[261] Tegeder I., Thiel K., Erkek S., Johann P.D., Berlandi J., Thatikonda V., Frühwald M.C., Kool M., Jeibmann A., Hasselblatt M. (2019). Functional relevance of genes predicted to be affected by epigenetic alterations in atypical teratoid/rhabdoid tumors. J. Neurooncol..

[262] Zhu S., Xu Y., Song M., Chen G., Wang H., Zhao Y., Wang Z., Li F. (2016). PRDM16 is associated with evasion of apoptosis by prostatic cancer cells according to RNA interference screening. Mol. Med. Rep..

[263] Singh R., Parveen M., Basgen J.M., Fazel S., Meshesha M.F., Thames E.C., Moore B., Martinez L., Howard C.B., Vergnes L. (2016). Increased Expression of Beige/Brown Adipose Markers from Host and Breast Cancer Cells Influence Xenograft Formation in Mice. Mol. Cancer Res..

[264] Elattar S., Dimri M., Satyanarayana A. (2018). The tumor secretory factor ZAG promotes white adipose tissue browning and energy wasting. FASEB J..

[265] Zhang H., Zhu L., Bai M., Liu Y., Zhan Y., Deng T., Yang H., Sun W., Wang X., Zhu K. (2019). Exosomal circRNA derived from gastric tumor promotes white adipose browning by targeting the miR-133/PRDM16 pathway. Int. J. Cancer.

[266] Avila-Fernandez A., Perez-Carro R., Corton M., Lopez-Molina M.I., Campello L., Garanto A., Fernandez-Sanchez L., Duijkers L., Lopez-Martinez M.A., Riveiro-Alvarez R. (2015). Whole-exome sequencing reveals ZNF408 as a new gene associated with autosomal recessive retinitis pigmentosa with vitreal alterations. Hum. Mol. Genet..

[267] Tsang A.P., Visvader J.E., Turner C.A., Fujiwara Y., Yu C., Weiss M.J., Crossley M., Orkin S.H. (1997). FOG, a multitype zinc finger protein, acts as a cofactor for transcription factor GATA-1 in erythroid and megakaryocytic differentiation. Cell.

[268] Tsang A.P., Fujiwara Y., Hom D.B., Orkin S.H. (1998). Failure of megakaryopoiesis and arrested erythropoiesis in mice lacking the GATA-1 transcriptional cofactor FOG. Genes Dev..

[269] Marcucci G., Maharry K., Radmacher M.D., Mrózek K., Vukosavljevic T., Paschka P., Whitman S.P., Langer C., Baldus C.D., Liu C.G. (2008). Prognostic significance of, and gene and microRNA expression signatures associated with, CEBPA mutations in cytogenetically normal acute myeloid leukemia with high-risk molecular features: A Cancer and Leukemia Group B Study. J. Clin. Oncol..

[270] Buck I., Morceau F., Cristofanon S., Reuter S., Dicato M., Diederich M. (2009). The inhibitory effect of the proinflammatory cytokine TNFalpha on erythroid differentiation involves erythroid transcription factor modulation. Int. J. Oncol..

[271] Yang H., Hui H., Wang Q., Li H., Zhao K., Zhou Y., Zhu Y., Wang X., You Q., Guo Q. (2014). Wogonin induces cell cycle arrest and erythroid differentiation in imatinib-resistant K562 cells and primary CML cells. Oncotarget.

[272] Cai Q., Jeannet R., Hua W.K., Cook G.J., Zhang B., Qi J., Liu H., Li L., Chen C.C., Marcucci G. (2016). CBFβ-SMMHC creates aberrant megakaryocyte-erythroid progenitors prone to leukemia initiation in mice. Blood.

[273] Fujiwara T., Sasaki K., Saito K., Hatta S., Ichikawa S., Kobayashi M., Okitsu Y., Fukuhara N., Onishi Y., Harigae H. (2017). Forced FOG1 expression in erythroleukemia cells: Induction of erythroid genes and repression of myelo-lymphoid transcription factor PU.1. Biochem. Biophys. Res. Commun..

[274] Liu A., Li S., Donnenberg V., Fu J., Gollin S.M., Ma H., Lu C., Stolz D.B., Mapara M.Y., Monaghan S.A. (2018). Immunomodulatory drugs downregulate IKZF1 leading to expansion of hematopoietic progenitors with concomitant block of megakaryocytic maturation. Haematologica.

[275] Litchfield K., Holroyd A., Lloyd A., Broderick P., Nsengimana J., Eeles R., Easton D.F., Dudakia D., Bishop D.T., Reid A. (2015). Identification of four new susceptibility loci for testicular germ cell tumour. Nat. Commun..

[276] Liu H., Zhao H. (2019). Prognosis related miRNAs, DNA methylation, and epigenetic interactions in lung adenocarcinoma. Neoplasma.

[277] Rahane C.S., Kutzner A., Heese K. (2019). Establishing a human adrenocortical carcinoma (ACC)-specific gene mutation signature. Cancer Genet..

[278] Cantor A.B., Orkin S.H. (2005). Coregulation of GATA factors by the Friend of GATA (FOG) family of multitype zinc finger proteins. Semin. Cell. Dev. Biol..

[279] Laitinen M.P., Anttonen M., Ketola I., Wilson D.B., Ritvos O., Butzow R., Heikinheimo M. (2000). Transcription factors GATA-4 and GATA-6 and a GATA family cofactor, FOG-2, are expressed in human ovary and sex cord-derived ovarian tumors. J. Clin. Endocrinol. Metab..

[280] Efimenko E., Padua M.B., Manuylov N.L., Fox S.C., Morse D.A., Tevosian S.G. (2013). The transcription factor GATA4 is required for follicular development and normal ovarian function. Dev. Biol..

[281] Anttonen M., Unkila-Kallio L., Leminen A., Butzow R., Heikinheimo M. (2005). High GATA-4 expression associates with aggressive behavior, whereas low anti-Müllerian hormone expression associates with growth potential of ovarian granulosa cell tumors. J. Clin. Endocrinol. Metab..

[282] Virgone C., Cecchetto G., Ferrari A., Bisogno G., Donofrio V., Boldrini R., Collini P., Dall’Igna P., Alaggio R. (2012). GATA-4 and FOG-2 expression in pediatric ovarian sex cord-stromal tumors replicates embryonal gonadal phenotype: Results from the TREP project. PLoS ONE.

[283] Salonen J., Rajpert-De Meyts E., Mannisto S., Nielsen J.E., Graem N., Toppari J., Heikinheimo M. (2010). Differential developmental expression of transcription factors GATA-4 and GATA-6, their cofactor FOG-2 and downstream target genes in testicular carcinoma in situ and germ cell tumors. Eur. J. Endocrinol..

[284] Manuylov N.L., Smagulova F.O., Tevosian S.G. (2007). Fog2 excision in mice leads to premature mammary gland involution and reduced Esr1 gene expression. Oncogene.

[285] Aumsuwan P., Khan S.I., Khan I.A., Ali Z., Avula B., Walker L.A., Shariat-Madar Z., Helferich W.G., Katzenellenbogen B.S., Dasmahapatra A.K. (2016). The anticancer potential of steroidal saponin, dioscin, isolated from wild yam (Dioscorea villosa) root extract in invasive human breast cancer cell line MDA-MB-231 in vitro. Arch. Biochem. Biophys..

[286] Wu H.C., Cohn B.A., Cirillo P.M., Santella R.M., Terry M.B. (2019). DDT exposure during pregnancy and DNA methylation alterations in female offspring in the Child Health and Development Study. Reprod. Toxicol..

[287] Hoene V., Fischer M., Ivanova A., Wallach T., Berthold F., Dame C. (2009). GATA factors in human neuroblastoma: Distinctive expression patterns in clinical subtypes. Br. J. Cancer.

[288] Tsang S.Y., Mei L., Wan W., Li J., Li Y., Zhao C., Ding X., Pun F.W., Hu X., Wang J. (2015). Glioma Association and Balancing Selection of ZFPM2. PLoS ONE.

[289] Vastrad B., Vastrad C., Godavarthi A., Chandrashekar R. (2017). Molecular mechanisms underlying gliomas and glioblastoma pathogenesis revealed by bioinformatics analysis of microarray data. Med. Oncol..

[290] Guan D., Tian H. (2017). Integrated network analysis to explore the key genes regulated by parathyroid hormone receptor 1 in osteosarcoma. World J. Surg. Oncol..

[291] Panagopoulos I., Gorunova L., Davidson B., Heim S. (2015). Novel TNS3-MAP3K3 and ZFPM2-ELF5 fusion genes identified by RNA sequencing in multicystic mesothelioma with t(7;17)(p12;q23) and t(8;11)(q23;p13). Cancer Lett..

[292] Karlsson J., Holmquist Mengelbier L., Elfving P., Gisselsson Nord D. (2011). High-resolution genomic profiling of an adult Wilms’ tumor: Evidence for a pathogenesis distinct from corresponding pediatric tumors. Virchows Arch..

[293] Kong F., Deng X., Kong X., Du Y., Li L., Zhu H., Wang Y., Xie D., Guha S., Li Z. (2018). ZFPM2-AS1, a novel lncRNA, attenuates the p53 pathway and promotes gastric carcinogenesis by stabilizing MIF. Oncogene.

[294] Yan J., Zhou C., Guo K., Li Q., Wang Z. (2019). A novel seven-lncRNA signature for prognosis prediction in hepatocellular carcinoma. J. Cell Biochem..

[295] Choi S.H., Ruggiero D., Sorice R., Song C., Nutile T., Vernon Smith A., Concas M.P., Traglia M., Barbieri C., Ndiaye N.C. (2016). Six Novel Loci Associated with Circulating VEGF Levels Identified by a Meta-analysis of Genome-Wide Association Studies. PLoS Genet..

[296] Clara J.A., Monge C., Yang Y., Takebe N. (2019). Targeting signalling pathways and the immune microenvironment of cancer stem cells—A clinical update. Nat. Rev. Clin. Oncol..

[297] Qin H., Ruan Z.H. (2014). The role of monoacylglycerol lipase (MAGL) in the cancer progress. Cell Biochem. Biophys..

[298] Allali-Hassani A., Szewczyk M.M., Ivanochko D., Organ S.L., Bok J., Ho J.S.Y., Gay F.P.H., Li F., Blazer L., Eram M.S. (2019). Discovery of a chemical probe for PRDM9. Nat. Commun..

[299] Wang B.D., Lee N.H. (2018). Aberrant RNA Splicing in Cancer and Drug Resistance. Cancers.

